# Liver enzyme elevations during statin–ezetimibe therapy in *ABCG2* c.421C>A variant carriers: a case series and narrative review

**DOI:** 10.3389/fphar.2026.1840055

**Published:** 2026-10-05

**Authors:** Livija Šimičević, Lana Ganoci, Ena Karačić, Tamara Božina, Ivan Pećin, Vladimir Trkulja, Nada Božina

**Affiliations:** 1 Division for Pharmacogenomics and Therapy Individualization, Department of Laboratory Diagnostics, University Hospital Centre Zagreb, Zagreb, Croatia; 2 Department for Chemistry and Biochemistry, University of Zagreb School of Medicine, Zagreb, Croatia; 3 Department of Basic and Clinical Pharmacology, Zagreb University School of Medicine, Zagreb, Croatia; 4 University of Zagreb Faculty of Pharmacy and Biochemistry, Zagreb, Croatia; 5 Division for Metabolic Diseases, Department of Internal Medicine, University Hospital Centre Zagreb, Zagreb, Croatia; 6 Department of Internal Medicine, University of Zagreb School of Medicine, Zagreb, Croatia

**Keywords:** ABCG2, adverse drug reactions, drug transporters, drug-drug-gene interactions, liver enzyme elevation, pharmacogenetics, pharmacokinetics, statin-ezetimibe combination

## Abstract

**Introduction:**

Statin–ezetimibe combination therapy is widely used and generally well tolerated; however, liver enzyme elevations may occur during treatment and can be difficult to interpret in patients with polypharmacy and other contributing factors. The potential role of drug–drug–gene interactions (DDGIs) in this context remains insufficiently explored.

**Materials and Methods:**

We present a series of 11 patients from the PGx-CardioDrug study, selected for documented liver enzyme elevations during statin–ezetimibe therapy that led to treatment modification and carriage of the *ABCG2* c.421A allele. Clinical characteristics, treatment history, laboratory findings, and outcomes were reviewed alongside available pharmacogenetic results for genes involved in drug transport and metabolism. Available records of five additional *ABCG2* c.421CA carriers with documented normal liver enzyme findings during combination therapy were reviewed for descriptive comparison. A narrative review of the literature was conducted to provide a mechanistic interpretation for the observed findings.

**Results:**

The series included six women and five men, aged 27–78 years. Muscle symptoms and/or creatine kinase (CK) elevations were observed in six patients, including two with rhabdomyolysis requiring hospitalization. Dose reduction, drug discontinuation, or switching to alternative lipid-lowering therapy resulted in improvement of laboratory parameters in several patients. In two patients, abnormalities persisted or increased during subsequent ezetimibe monotherapy. Follow-up laboratory results were not available for all patients. Eight patients had the *ABCG2* c.421CA genotype and three had c.421AA, consistent with the selection criterion. Additional findings included variants in *SLCO1B1*, *ABCC2*, *ABCB1*, and *UGT1A1*. In the five potential comparators, normal liver enzyme findings during combination therapy were supported by numerical laboratory values in three patients and clinical documentation in two.

**Discussion:**

These cases illustrate the difficulty of interpreting liver enzyme elevations during statin–ezetimibe therapy in patients with concomitant medications and other contributing factors. The narrative review provides a pharmacokinetic rationale for considering DDGIs, but the case series does not establish their contribution to the observed abnormalities. Genotype-based selection, incomplete clinical data, and the small descriptive comparator group limit interpretation. These observations remain hypothesis-generating.

## 1 Introduction

Hydroxymethylglutaryl-CoA reductase inhibitors (statins) remain the cornerstone of lipid-lowering therapy and the first-line treatment for hypercholesterolemia in patients at high cardiovascular risk (Mach et al., 2025; Lloyd-Jones et al., 2022; Rao et al., 2025). By inhibiting the rate-limiting enzyme of cholesterol biosynthesis, statins effectively reduce plasma levels of total cholesterol and low-density lipoprotein cholesterol (LDL-C) concentrations (Preiss et al., 2020).

With increasingly strict LDL-C targets recommended by contemporary guidelines, combination lipid-lowering therapy has become increasingly common in clinical practice (Mach et al., 2025; Lloyd-Jones et al., 2022). Ezetimibe is among the most frequently used agents added to statin therapy when LDL-C targets are not achieved with statin treatment alone. Both drugs are generally well tolerated and current evidence suggests that low- or moderate-intensity statin plus ezetimibe may result in fewer muscle-related events and liver enzyme elevations than high-intensity statin monotherapy (Sydhom et al., 2024). However, adverse drug reactions (ADRs) can still occur in individual patients, and may lead to treatment discontinuation or poor adherence (Bradley et al., 2019; Ward et al., 2019).

The most commonly reported ADRs associated with statin therapy are muscle-related, ranging from mild myalgia to rare but severe complications of rhabdomyolysis (<0.1%) (Reith et al., 2022). Liver enzyme elevations are less frequent and are usually mild and transient, whereas clinically significant liver injury is rare (≈0.001%) (Licata et al., 2019; Newman et al., 2019; Preiss et al., 2020). However, liver enzyme abnormalities remain relevant because they may trigger treatment modification or discontinuation and can be difficult to attribute to a single cause in patients receiving combination therapy.

Statins have been extensively studied in the field of pharmacogenetics, and variants in drug-metabolizing enzymes and membrane transporters contribute to interindividual variability in statin pharmacokinetics, efficacy and ADRs (Link, 2008; Guan et al., 2019; Turner et al., 2020b; Cooper-DeHoff et al., 2022; Mykkänen et al., 2022; Lönnberg et al., 2023; Swen et al., 2023;Hoste et al., 2024; Wolthuis et al., 2025). Clinical pharmacogenetic guidelines initially focused on the association between *SLCO1B1* variants and simvastatin-induced myotoxicity and have subsequently expanded to other statins and pharmacogenes including ABCG2 and CYP2C9 (Wilke et al., 2012; Wolthuis et al., 2025; Cooper-DeHoff et al., 2022). However, the pharmacogenetic determinants of statin-associated liver injury remain poorly characterized (Licata et al., 2019; Liu et al., 2019).

Ezetimibe inhibits the cholesterol uptake transporter Niemann–Pick C1-like protein 1 (NPC1L1) in the small intestine and liver (Huang et al., 2020; Preiss et al., 2020). Its major metabolic pathway involves glucuronidation by UDP-glucuronosyltransferases (UGTs), resulting in formation of ezetimibe glucuronide, an active metabolite accounting for 80%–90% of total circulating drug (Ghosal et al., 2004; Kosoglou et al., 2005; Oswald et al., 2006; Yamamoto et al., 2007; Soulele and Karalis, 2019). Ezetimibe undergoes extensive first-pass metabolism in intestinal cells, followed by enterohepatic recirculation involving several transporters, including P-glycoprotein (ABCB1/MDR1), organic anion transporting polypeptide 1B1 (OATP1B1/SLCO1B1), multidrug resistance protein 2 (ABCC2/MRP2), and breast cancer resistance protein (ABCG2/BCRP) (Oswald et al., 2006; De Waart et al., 2009; Su et al., 2025). Although ezetimibe is generally considered safe, sporadic cases of liver injury have been reported during both monotherapy and combination therapy with statins (Kashani et al., 2008; Toth et al., 2012; Preiss et al., 2020).

Pharmacogenetic studies of ezetimibe pharmacokinetics and response have identified associations with variants in genes encoding drug transporters and metabolic enzymes, including *ABCB1*, *ABCC2*, *ABCG5*, *ABCG8*, *NPC1L1* and *UGT1A1* (Hegele et al., 2005; Oswald et al., 2008; Bae et al., 2011; Tada et al., 2020; González-Iglesias et al., 2024). However, these findings have not yet been translated into clinically applicable pharmacogenetic guidelines.

Drug–drug interactions (DDIs) and drug–gene interactions (DGIs) represent additional sources of variability in drug response and susceptibility to ADRs. Pharmacokinetic DDIs may involve cytochrome P450 (CYP) and UGT enzymes, as well as ATP-binding cassette (ABC) and solute carrier (SLC) transporters, several of which are polymorphic and shared by statins and ezetimibe (Pirmohamed, 2023; Delabays et al., 2024; Božina and Stingl, 2025). The convergence of genetic variability and concomitant drug exposure within shared pharmacokinetic pathways provides the basis for drug–drug–gene interactions (DDGIs), which may be particularly relevant in patients receiving combination lipid-lowering therapy and multiple co-medications. In this paper, we present a series of 11 patients selected for liver enzyme elevations during statin–ezetimibe therapy and carriage of the *ABCG2* c.421A allele. We integrate these clinical observations with a narrative review of the pharmacogenetic and pharmacokinetic evidence relevant to statin–ezetimibe disposition. Available records of five additional *ABCG2* c.421CA carriers with normal liver enzyme findings during combination therapy were reviewed for descriptive comparison. Particular attention is given to shared transporter and metabolic pathways and to the potential role of DDGIs as a framework for interpreting interindividual variability in treatment tolerability.

## 2 Materials and methods

### 2.1 Patients

The presented patients were identified within a larger prospective pharmacogenomic study [Pharmacogenomics in Prediction of Cardiovascular Drugs Adverse Reaction—PGx CardioDrug (NCT05307718)], which investigated the role of pharmacogenetic factors in predicting adverse drug reactions to cardiovascular medications (Božina et al., 2026).

Between May 2021 and May 2025, 1,965 patients treated with statins were included in the PGx-CardioDrug study. Among these patients, concomitant ezetimibe use has been documented in 204 patients to date. Review of treatment records is ongoing, and additional patients receiving combination therapy may be identified.

For the present case series, patients were identified during clinical follow-up within the PGx-CardioDrug study and subsequent review of medical records. We included 11 patients with documented alanine aminotransferase (ALT) and/or aspartate aminotransferase (AST) elevations during statin–ezetimibe therapy, modification of lipid-lowering treatment following these findings, and at least one *ABCG2* c.421A allele (Figure 1). Muscle symptoms and creatine kinase (CK) elevations were present in some patients but were not required for inclusion.

**FIGURE 1:**
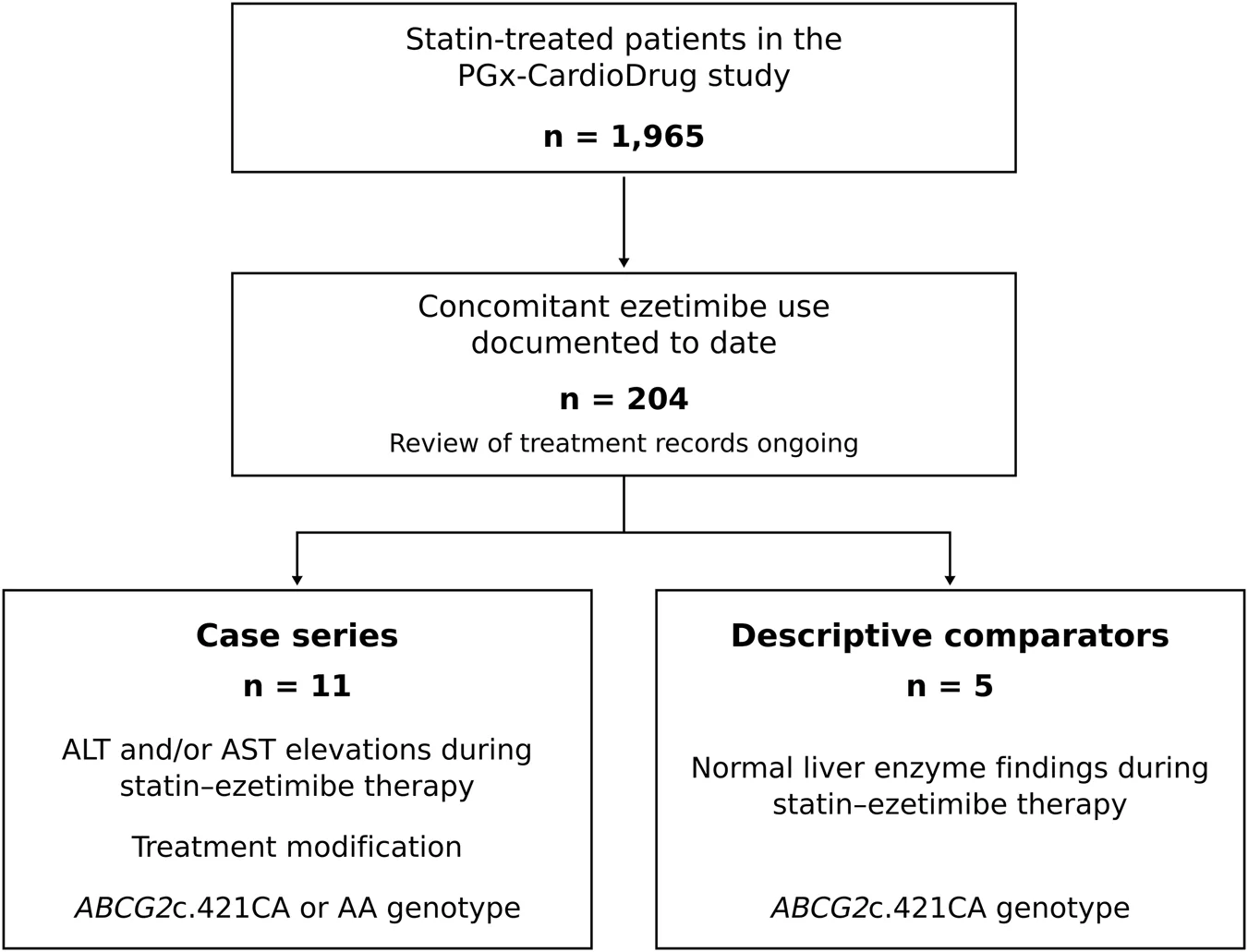
Identification of cases and comparator patients within the PGx-CardioDrug study. Concomitant ezetimibe use has been documented in 204 patients to date. Review of treatment records and assessment of eligibility for this case series are incomplete; the numbers shown do not represent exhaustive screening of the cohort.

A detailed review of the entire cohort for these criteria has not been completed. Therefore, the series may not include all eligible patients and cannot be used to estimate the frequency of liver enzyme elevations or genotype-associated risk. Five additional patients with the *ABCG2* c.421CA genotype and documented normal liver enzyme levels during combination therapy were included for descriptive comparison.

Details of the overall recruitment and follow-up procedures have been reported previously (Božina et al., 2026).

### 2.2 Pharmacogenetic analysis

Pharmacogenetic testing focused on genes involved in drug metabolism and transport that are relevant for statin and ezetimibe pharmacokinetics. Variants were selected based on previous literature evidence of their association with drug disposition, treatment response, or the risk of adverse drug reactions.

Genomic DNA was extracted from 3 mL of peripheral blood collected in EDTA tubes using the QIAamp DNA Mini Kit (Qiagen, Hilden, Germany), according to the manufacturer’s instructions. Genotyping was performed on 7500 Real-Time PCR System (Applied Biosystems, Foster City, CA, United States) using specific TaqMan® DME Assay and TaqMan® SNP Assay reagents (Applied Biosystems, Foster City, CA, United States). Positive and negative controls were included in each analytical run. Samples with inconclusive genotyping results were reanalysed.

Pharmacogenetic results presented in this report include: *CYP2C9* (*2, *3), *CYP2C19* (*2, *17), *CYP3A4* (*1B, *22), CYP3A5*3, *ABCB1* (c.1236C>T, c.2677G>T/A, c.3435C>T), *ABCC2* (c.-24C>T, c.1249G>A), *ABCG2* (c.34G>A, c.421C>A), *SLCO1B1* (c.388A>G, c.521T>C, c.463C>A, c.1929A>C), N-acetyltransferase 2 gene (*NAT2*) (*5, *6, *7), *UGT1A1* (*28), and *UGT2B7* c. −161 C>T. Results were not available for all listed variants in every patient. Available pharmacogenetic results are presented in Supplementary Table S1. Missing results are indicated as not reported (NR) and were not assumed to represent reference genotypes. Star allele nomenclature was used where applicable. Transporter variants were reported as individual nucleotide genotypes. *SLCO1B1* diplotypes discussed in the text were inferred from the available variant results where these supported their assignment. Where a diplotype could not be assigned unambiguously, individual variant genotypes were reported.

Pharmacogenetic testing was performed at the Department of Laboratory Diagnostics, University Hospital Centre Zagreb. The department is accredited according to HRN EN ISO 15189:2022 and participates in external quality assessment programmes provided by Foundation for Pathobiochemistry and Molecular Diagnostics–Reference Institute for Bioanalytics (SPMD-RfB) and European Molecular Genetics Quality Network (EMQN).

### 2.3 Definition and classification of clinical and laboratory abnormalities

Clinical symptoms and laboratory abnormalities documented during statin–ezetimibe therapy were classified descriptively. These categories describe the observed clinical phenotype and do not, by themselves, establish a causal relationship with treatment. Muscle-related findings were classified as follows.

- Myalgia–muscle pain or discomfort with CK within the laboratory reference range.
- Mild CK elevation–increase in CK levels more than one time (>1) and less than three times (<3x) the upper limit of normal (ULN).
- Myotoxicity–increase in CK levels ≥3x ULN.

The CK categories were used as descriptive thresholds for this case series rather than as diagnostic criteria for drug-induced myotoxicity. The presence or absence of accompanying muscle symptoms was considered separately.

Liver enzyme elevations were defined as increase in alanine aminotransferase (ALT) and/or aspartate aminotransferase (AST) above the ULN during therapy.

- For the assessment of potential liver injury, the following biochemical thresholds were considered: ALT ≥5× ULN, alkaline phosphatase (ALP) ≥2× ULN, or ALT ≥3× ULN accompanied by total bilirubin >2× ULN. In patients with pre-existing liver test abnormalities, changes from baseline values were also considered (European Association for the Study of the Liver, 2019).

These thresholds were distinguished from lesser aminotransferase elevations and were not considered sufficient to establish drug-induced liver injury (DILI).

ALP measurements were available for some patients, but the laboratory data required to assess the ALP-based criterion and calculate the R ratio were not consistently available across the series. Classification of the biochemical pattern as hepatocellular, cholestatic, or mixed was therefore limited by the available data. Missing laboratory measurements were not assumed to be normal.

DILI was not assigned solely on the basis of biochemical abnormalities, as attribution to a specific drug requires consideration of the temporal relationship, alternative causes, concomitant medications, and clinical course following treatment withdrawal and, where applicable, re-exposure.

### 2.4 Assessment of causality and potential drug interactions

For each patient, we reviewed the timing of laboratory abnormalities in relation to treatment, the course after treatment modification, and potential alternative contributors, including comorbidities and concomitant medications. Causality was assessed descriptively. Roussel Uclaf Causality Assessment Method (RUCAM) scores were not calculated because the information required for reliable scoring was not consistently available. Documented pharmacokinetic interactions were distinguished from proposed mechanisms; neither was considered proof that an interaction caused the abnormalities in an individual patient. Patient-level assessments are presented in Supplementary Table S2. Clinical characteristics and available pharmacogenetic results of the comparator patients are provided in Supplementary Table S3. Available laboratory findings before, during, and after treatment modification are summarised in Supplementary Table S4.

### 2.5 Ethical considerations

The study was conducted in accordance with the principles of the Declaration of Helsinki. The study protocol was approved by the institutional ethics committee. Written informed consent was obtained from all participants prior to inclusion in the study.

## 3 Results

The present series included 11 patients (six women and five men), aged 27–78 years, with clinical and laboratory abnormalities documented during statin–ezetimibe therapy.

Six patients had muscle symptoms and/or elevated CK levels (Cases 1, 3, 7, 8, 9, and 11). Two patients developed rhabdomyolysis (Cases 8 and 9). In Case 8, rhabdomyolysis was accompanied by acute kidney injury requiring hospitalization. Patient-level clinical characteristics and laboratory findings are summarized in Table 1.

**TABLE 1:** Clinical characteristics, lipid-lowering therapy, clinical and laboratory abnormalities, and selected pharmacogenetic findings in 11 patients included in the case series.

Case	Sex	Age	Lipid-lowering therapy	Clinical and laboratory abnormalities	Peak ALT (U/L)	Peak AST (U/L)	Peak CK (U/L)	Key pharmacogenetic variants	Therapy modification
1	M	51	**Atorvastatin (20 mg/day) →** **Rosuvastatin (5 mg/day) +** **Ezetimibe (10 mg/day) →** **Ezetimibe (10 mg/day)** → Atorvastatin (10 mg/day)	Myotoxicity and aminotransferase elevations	114	57	1,700	*ABCG2* c.421C**A** *ABCC2* c.1249G**A** UGT1A1 *1/* ***28*** *SLCO1B1* *1/*14	Statin withdrawal and switching; ezetimibe withdrawal; subsequent reintroduction of low-dose atorvastatin
2	M	57	**Rosuvastatin/ezetimibe (20/10 mg/day)** → both drugs discontinued Lp(a) apheresis initiated→ **Rosuvastatin**/**ezetimibe** (**40/10 mg/day)** reintroduced 1 year later → alirocumab added 1 year thereafter	Liver enzyme elevations in the context of underlying liver disease	140	184	—	*ABCG2* c.421**AA** *ABCC2* c.1249G**A** *ABCB1* c.1236**TT,** c.2677**TT,** c.3435**TT** SLCO1B1 *1/* ***5*** UGT1A1 *1/* ***28***	Rosuvastatin and ezetimibe discontinued and subsequently reintroduced; lipoprotein apheresis initiated; alirocumab subsequently added
3	M	43	**Rosuvastatin (20 mg/day) →** **Rosuvastatin + ezetimibe (20/10 mg/day)** → Atorvastatin (40 mg/day) + ezetimibe (10 mg, every other day)	Myalgia, myotoxicity, and aminotransferase elevations	91	60	1,069	*ABCG2* c.421 ***AA*** *SLCO1B1* *1/*37	Statin switching; reduced ezetimibe dosing frequency
4	F	58	**Atorvastatin + ezetimibe (40/10 mg/day)** → Ezetimibe (10 mg/day) → Alirocumab	Aminotransferase and marked GGT elevations	123	219	—	*ABCG2* c.421C ***A*** *ABCC2* c.1249G ***A*** *ABCB1* c.1236**TT,** c.2677**TT,** c.3435**TT** SLCO1B1 *1/* ***15*** UGT1A1 *1/* ***28***	Statin withdrawn; ezetimibe withdrawn; PCSK9 inhibitor
5	F	68	Rosuvastatin (40 mg/day) → **Rosuvastatin + ezetimibe (40/10 mg/day)** → Rosuvastatin (20 mg/day) → Atorvastatin (80 mg/day) → Atorvastatin (40 mg/day) → alirocumab	Liver enzyme elevations	93	57	—	*ABCG2* c.421C ***A*** *ABCC2* c.1249G ***A***	Ezetimibe withdrawn; statin dose reduction and switching; subsequent statin withdrawal and switch to PCSK9 inhibitor
6	F	62	**Rosuvastatin + ezetimibe (20/10 mg/day)** → Ezetimibe (10 mg/day) → Ezetimibe (10 mg/day) + evolocumab → Ezetimibe (10 mg/day) + evolocumab; addition of atorvastatin (10 mg, every third day)	Aminotransferase elevations, including ALT ≥5× ULN	204	79	—	*ABCG2* c.421C ***A*** *ABCC2* c.1249G ***A*** *ABCB1* c.1236**TT,** c.2677**TT,** c.3435**TT** UGT1A1 *1/* ***28*** *SLCO1B1* *1/*37	Rosuvastatin withdrawn; ezetimibe continued; evolocumab added; low-dose atorvastatin recommended
7	F	27	**Rosuvastatin + ezetimibe (20/10 mg/day)** → **Rosuvastatin + ezetimibe (5/10 mg/day) →** **Atorvastatin + ezetimibe (20/10 mg/day)** → Fluvastatin (80 mg/day) + ezetimibe (10 mg), subsequently reduced to every other day and then every third day	Myalgia and abdominal pain; mild ALT elevation	53	25	—	*ABCG2* c.421**AA** *ABCC2* c.1249**AA** *ABCB1* c.1236**TT,** c.2677**TT,** c.3435C**T** CYP3A4 *1/* ***22*** *SLCO1B1* *1/* ***37***	Statin dose reduction and switching; reduced ezetimibe dosing frequency
8	F	76	Rosuvastatin (10 mg/day) → **Rosuvastatin + ezetimibe (40/10 mg/day**)	Rhabdomyolysis with acute kidney injury and marked aminotransferase elevations	773	1,206	11,837	*ABCG2* c.421C ***A*** *ABCC2* c.1249G ***A*** *SLCO1B1* *1/ *15 *UGT1A1* *28/*28 *UGT2B7* c.-161**TT**	Rosuvastatin withdrawn; ezetimibe withdrawn
9	F	78	**Rosuvastatin + ezetimibe** fixed-dose combination (dose unknown)	Rhabdomyolysis requiring hospitalization, accompanied by ALT elevation	239	—	6,704	*ABCG2 c.*421C**A*;*** *SLCO1B1 c.*521T**C** and *c.*463C**A**	Rosuvastatin–ezetimibe combination discontinued; switched to ezetimibe monotherapy (10 mg/day)
10	M	31	**Rosuvastatin (20 mg/day) + ezetimibe (10 mg/day) →** **Rosuvastatin (10 mg/day) + ezetimibe (10 mg/day) →** **Rosuvastatin (5 mg/day) + ezetimibe (10 mg/day)**	Liver enzyme elevations	133	49	—	*ABCG2 *c.421C**A**	Rosuvastatin reduced from 20 to 10 mg/day, and subsequently to 5 mg/day; ezetimibe continued at 10 mg/day
11	M	48	**Rosuvastatin (10 mg/day) + ezetimibe (10 mg/day) → ezetimibe (10 mg/day)**	Elevated CK and aminotransferases, without muscle pain or weakness	83^a^	148^a^	5,466^a^	*ABCG2 *c.421C**A** *; SLCO1B1 *c.521T**C**	Rosuvastatin discontinued; subsequent ezetimibe withdrawal recommended after further CK elevation. Follow-up laboratory results were not available

The table summarizes demographic characteristics, lipid-lowering therapy, clinical and laboratory abnormalities, peak laboratory values and key pharmacogenetic variants identified in the analysed pharmacogenes. Peak values for individual laboratory parameters may have been recorded at different visits. A dash indicates that a numerical peak value is not reported, rather than indicating a normal result. *ABCB1* variants are reported individually as genotypes for c.1236C>T (rs1128503), c.2677G>T/A (rs2032582), and c.3435C>T (rs1045642).

Clinical and laboratory abnormalities were classified descriptively. Clinically significant liver enzyme elevation was defined as ALT ≥5× ULN, ALP ≥2× ULN, or ALT ≥3× ULN accompanied by total bilirubin >2× ULN (European Association for the Study of the Liver, 2019). These thresholds alone did not establish drug-induced liver injury. CK, elevation ≥3× ULN, was used as a descriptive threshold for muscle enzyme elevation.

Reference intervals used in the laboratory were as follows: for men (M), ALT 12–48 U/L, AST 11–38 U/L, and CK 0–177 U/L; for women (F), ALT 10–36 U/L, AST 8–30 U/L, and CK 0–153 U/L.

Bold type indicates the presence of specific polymorphisms and the corresponding therapy during which the adverse event occurred.

Abbreviations: ALT, alanine aminotransferase; AST, aspartate aminotransferase; ALP, alkaline phosphatase; CK, creatine kinase; GGT, gamma-glutamyl transferase; ADR, adverse drug reaction; *SLCO1B1*, solute carrier organic anion transporter family member 1B1; *ABCG2*, ATP-binding cassette subfamily G member 2; *ABCB1*, ATP-binding cassette subfamily B member 1; *ABCC2*, ATP-binding cassette subfamily C member 2; *UGT*, UDP-glucuronosyltransferase; ULN, upper limit of normal; Lp(a), lipoprotein(a); PCSK9, proprotein convertase subtilisin/kexin type 9; CYP, cytochrome P450.

Detailed genotyping results are provided in Supplementary Table S1.

^a^ Peak values were recorded during ezetimibe monotherapy. During combination therapy, ALT was 69 U/L, AST 62 U/L, and CK 1,244 U/L.

Pharmacogenetic analysis revealed variants in genes involved in statin and ezetimibe transport and metabolism. Eight patients had the *ABCG2* c.421CA genotype and three had the AA genotype. The *SLCO1B1* c.521TC genotype was present in five patients, while six had the TT genotype. Additional variants were identified in *ABCC2*, *ABCB1*, *CYP3A4/5*, and *UGT1A1* (Table 1). Detailed pharmacogenetic results are provided in Supplementary Table S1.

Relevant comorbidities, concomitant medications, potential contributing factors, and limitations of causality assessment are summarised for each patient in Supplementary Table S2. Available laboratory findings and the course after treatment modification are presented in Supplementary Table S4.

The five comparator patients (two women and three men), aged 47–68 years, had the *ABCG2* c.421CA genotype and normal liver enzyme levels documented during statin–ezetimibe therapy. Four patients received rosuvastatin (20–40 mg/day) and one atorvastatin (80 mg/day), all with ezetimibe 10 mg/day. Clinical and laboratory data, concomitant therapy, and available genotypes are provided in Supplementary Table S3.

### 3.1 Case 1

A 51-year-old man with dyslipidemia, hypertension, and type 2 diabetes mellitus developed elevation in liver enzyme and CK approximately 11 months after starting atorvastatin (20 mg/day), causing treatment discontinuation. Concomitant medications included esomeprazole, nebivolol, spironolactone, perindopril/indapamide/amlodipine, metformin, meloxicam, dexketoprofen/tramadol, acetaminophen/tramadol, amitriptyline, and alprazolam. Following partial improvement in liver enzyme levels, therapy was switched to rosuvastatin (5 mg/day) in combination with ezetimibe (10 mg/day). After 3 months, a further increase in enzyme levels was observed, with peak values of AST 57 U/L, ALT 114 U/L, gamma-glutamyl transferase (GGT) 94 U/L, and CK 1,700 U/L. Rosuvastatin was discontinued, while ezetimibe (10 mg/day) was continued as monotherapy, but liver enzyme elevations persisted during approximately 13 months of ezetimibe treatment. Ezetimibe was also discontinued. After approximately 5 months without lipid-lowering therapy, a low-dose atorvastatin (10 mg/day) was reintroduced, with liver enzyme levels remaining within the reference interval during follow-up. Pharmacogenetic testing identified the following variants and genotypes: *ABCG2* c.421C**A**, *ABCC2* c.1249G**A**, CYP2C19 * ***2*** /*17, *CYP3A5* *3/*3, NAT2 * ***6*** /* ***6***, UGT1A1 *1/* ***28***; and SLCO1B1 *1/* ***14*** (c.388AG, c.463CA).

### 3.2 Case 2

A 57-year-old man with dyslipidemia, coronary artery disease, elevated lipoprotein(a), and imaging findings suggestive of hepatic steatosis developed elevated liver enzymes 18 months after starting rosuvastatin (20 mg/day) and ezetimibe (10 mg/day): AST 78 U/L, ALT 120 U/L, and GGT 348 U/L. Previous episodes of liver enzyme elevation during atorvastatin and rosuvastatin treatment were also documented. Concomitant treatment included nebivolol, acetylsalicylic acid, furosemide, and pantoprazole. Rosuvastatin and ezetimibe were discontinued, and regular lipoprotein apheresis was initiated, after which liver enzyme levels decreased. After 1 year, rosuvastatin (40 mg/day) and ezetimibe (10 mg/day) were reintroduced, followed by the addition of alirocumab (150 mg once every 2 weeks) 1 year later. Four years after reintroduction of rosuvastatin and ezetimibe, further elevation of liver enzymes (AST 184 U/L, ALT 140 U/L, and GGT 375 U/L) were documented, together with chronic liver disease, Model for End-Stage Liver Disease–sodium (MELD-Na) score of 9. The underlying liver disease and the prolonged interval between treatment reintroduction and the later abnormalities limit attribution of these findings to lipid-lowering therapy. Pharmacogenetic testing identified the following variants and genotypes: *ABCG2* c.421**AA**, SLCO1B1 *1/*5, ABCB1 c.1236**TT**, *ABCB1* c.2677**TT**, *ABCB1* c.3435**TT**, *ABCC2* c.1249G**A**, CYP2C19 * ***2*** /*17, CYP3A5 * 3/*3, NAT2 *4/* ***6*** , UGT1A1 *1/* ***28***.

### 3.3 Case 3

A 43-year-old man with dyslipidemia developed markedly elevated CK levels (reaching up to ∼1,000 U/L) during rosuvastatin treatment (20 mg/day), with some elevations occurring in the context of regular physical activity. Ezetimibe (10 mg/day) was subsequently added. Available records did not indicate relevant concomitant medication. After 12 months of combination therapy, liver enzyme elevations (AST 60 U/L, and ALT 91 U/L) and elevated CK level (1,069 U/L) with myalgia were observed, even in the absence of recent physical exertion. Rosuvastatin was replaced with atorvastatin (40 mg/day) and the dosing frequency of ezetimibe was reduced to 10 mg every other day. During follow-up, CK levels fluctuated but gradually improved. Pharmacogenetic testing identified the following variants and genotypes: *ABCG2* c.**421AA,** CYP2C9 *1/* ***2*,** *CYP3A5* *3/*3, NAT2 *4/* ***5***, CYP2C19 *1/* ***17*** and SLCO1B1 *1/* ***37***.

### 3.4 Case 4

A 58-year-old woman with familial hypercholesterolemia and a history of myocardial infarction, coronary artery bypass grafting, and percutaneous coronary intervention developed liver enzyme elevations approximately 12 months after starting atorvastatin (40 mg/day) and ezetimibe (10 mg/day). Laboratory findings on 18 July 2022 were AST 213 U/L, ALT 123 U/L, and GGT 535 U/L. Concomitant medications included amlodipine, bisoprolol, acetylsalicylic acid, pantoprazole, diazepam, and alprazolam. Treatment was continued because of the patient’s high cardiovascular risk. Repeat testing on 7 September 2022 showed persistent abnormalities (AST 219 U/L, ALT 93 U/L, and GGT 1,048 U/L). Consequently, atorvastatin was discontinued, while ezetimibe was initially continued but was later replaced with a proprotein convertase subtilisin/kexin type 9 (PCSK9) inhibitor (alirocumab). Laboratory testing during follow-up showed lower AST and GGT levels, but persistent biochemical abnormalities (AST 80 U/L, ALT 98 U/L, and GGT 419 U/L). Pharmacogenetic testing identified the following variants and genotypes: *ABCG2* c.421C**A**, SLCO1B1 *1/* ***15*** *, ABCB1* c.1236**TT**, *ABCB1* c.2677**TT**, *ABCB1* c.3435**TT**, *ABCC2* c.1249G**A**, CYP2C9 *1/* ***2***, CYP3A5 *3/*3, NAT2 * ***6*** /* ***6*** , UGT1A1 *1/* ***28***.

### 3.5 Case 5

A 68-year-old woman with familial hypercholesterolemia and osteoporosis was initially treated with rosuvastatin (40 mg/day) and baseline liver enzyme levels were within reference intervals (AST 26 U/L, ALT 31 U/L, GGT 38 U/L). Concomitant medication included rabeprazole, citalopram, alprazolam, ibandronate, melatonin, and vitamin D. Approximately seven to 8 months after ezetimibe (10 mg/day) was added, mild liver enzyme elevations were observed (AST 57 U/L, ALT 93 U/L, and GGT 55 U/L). Consequently, ezetimibe was discontinued and the rosuvastatin dose reduced to 20 mg/day. Approximately 1 month after this change, laboratory testing on 9 October 2023 showed lower aminotransferase levels (AST 39 U/L, ALT 54 U/L, and GGT 44 U/L). During subsequent follow-up, rosuvastatin was replaced with atorvastatin (80 mg/day). In July 2025, further liver enzyme abnormalities were noticed (AST 39 U/L, ALT 57 U/L, and GGT 157 U/L), prompting a dose reduction of atorvastatin to 40 mg/day. Atorvastatin was later replaced with alirocumab (300 mg once every 4 weeks), after which liver enzymes normalised. Pharmacogenetic testing identified the following variants and genotypes: *ABCG2* c.421C**A**, *ABCC2* c.1249G**A**, CYP3A5 * 3/*3, NAT2 *4/* ***6***.

### 3.6 Case 6

A 62-year-old woman with dyslipidemia, hypertension, coronary and peripheral arterial disease, renal artery stenosis, and generalized atherosclerosis developed elevated liver transaminases approximately 3 months after starting rosuvastatin (20 mg/day) and ezetimibe (10 mg/day). Laboratory findings during this episode were AST 69 U/L, ALT 161 U/L and GGT 24 U/L, another measurement showed AST 79 U/L, ALT 204 U/L, and GGT 23 U/L. Concomitant medications included amlodipine/valsartan, nebivolol, ticagrelor, acetylsalicylic acid, and spironolactone. Rosuvastatin was discontinued because of suspected intolerance, while ezetimibe was continued. One year later, a PCSK9 inhibitor (evolocumab) was introduced to improve lipid control. During follow-up, the patient remained on evolocumab and ezetimibe, with a recommendation to add low-dose atorvastatin (10 mg every third day). Pharmacogenetic testing identified the following variants and genotypes: *ABCG2* c.421C**A**, *ABCB1* c.1236**TT**, *ABCB1* c.2677**TT**, *ABCB1* c.3435**TT**, *ABCC2* c.1249G**A**, CYP3A5 * 3/*3, NAT2 *4/* ***5*** , UGT1A1 *1/* ***28***, CYP2C19 *1/* ***17*** and SLCO1B1 *1/* ***37***.

### 3.7 Case 7

A 27-year-old woman with dyslipidemia and a history of smoking, developed myalgia and abdominal pain approximately 2 months after starting rosuvastatin (20 mg/day) and ezetimibe (10 mg/day), at that time liver enzyme and CK levels were within reference intervals. Available records did not indicate relevant concomitant medication. The rosuvastatin dose was reduced to 5 mg/day. Due to persistent muscle symptoms, rosuvastatin was replaced with atorvastatin (20 mg/day), while ezetimibe (10 mg/day) was continued. Myalgia recurred, prompting discontinuation of atorvastatin, after which the muscle symptoms resolved within 3 weeks. Therapy was subsequently changed to fluvastatin (80 mg/day) and ezetimibe (10 mg/day). During follow-up, a mild ALT elevation was noticed (ALT 53 U/L, AST 25 U/L, and GGT 37 U/L). The dosing frequency of ezetimibe was reduced to 10 mg every other day and later to every third day. Pharmacogenetic testing identified the following variants and genotypes: *ABCG2* c.421**AA**, *ABCB1* c.1236**TT**, *ABCB1* c.2677**TT**, *ABCB1* c.3435C**T**, *ABCC2* c.1249**AA**, CYP2C19 *1/* ***2***, CYP3A4 *1/* ***22***, *CYP3A5* *3/*3, NAT2 * ***5*** /* ***6***, and SLCO1B1 *1/* ***37***.

### 3.8 Case 8

A 76-year-old woman with dyslipidemia, hypertension, type 2 diabetes mellitus, paroxysmal atrial fibrillation, combined aortic valve disease, and a history of stroke had undergone right nephrectomy for an oncocytoma in 2014. Before the reported episode, renal function was preserved (creatinine 60 μmol/L, urea 4.9 mmol/L). Her lipid-lowering therapy was changed from rosuvastatin (10 mg/day) to a fixed-dose combination of rosuvastatin 40 mg and ezetimibe 10 mg daily. Concomitant medications included dapagliflozin/metformin, amlodipine/valsartan/hydrochlorothiazide, furosemide, valsartan, bisoprolol, alprazolam, apixaban, and levothyroxine, with tramadol/paracetamol taken as needed. Following this treatment change, the patient was hospitalised with acute kidney injury and marked aminotransferase elevations. Rosuvastatin, ezetimibe, apixaban, metformin, and dapagliflozin were discontinued. Initial laboratory findings showed markedly elevated creatinine (593 μmol/L) and urea (25.3 mmol/L), together with AST 517 U/L, ALT 323 U/L, and GGT 50 U/L. During hospitalization, renal function further deteriorated (creatinine 831 μmol/L, urea 30 mmol/L), accompanied by a pronounced increase in liver enzymes (AST 1,206 U/L, ALT 773 U/L, and GGT 62 U/L). Severe rhabdomyolysis was confirmed by elevated muscle injury markers (CK 11,837 U/L, myoglobin 49,100 μg/L), and hemodialysis was required. Imaging excluded obstructive causes of acute kidney injury, and immunological workup for inflammatory myopathies was negative. Statin-induced rhabdomyolysis was suspected on clinical grounds following escalation to high-dose rosuvastatin in combination with ezetimibe. Skeletal muscle injury may have contributed to the aminotransferase elevations; these findings alone did not establish a separate severe hepatocellular injury.

Pharmacogenetic testing identified the following variants and genotypes: *ABCG2* c.421C**A**, *ABCG2* c.34G**A**, *ABCC2* c.1249G**A**, SLCO1B1 *1/* ***15*** , CYP2C9 *1/* ***3***, *CYP3A5* *3/*3, NAT2 *4/* ***5***, UGT1A1 * ***28*** /* ***28***, *UGT2B7* c.-161**TT**.

### 3.9 Case 9

A 78-year-old woman with dyslipidemia, diabetes mellitus, hypertension, chronic kidney disease (estimated glomerular filtration rate [eGFR] <15 mL/min/1.73 m^2^), hemolytic anemia, hyperbilirubinemia, hyperuricemia, and generalized atherosclerotic disease developed rhabdomyolysis during treatment with a fixed-dose combination of rosuvastatin and ezetimibe. The combination dose and the interval between treatment initiation and the event were not documented. Concomitant medications included gliclazide, sitagliptin/metformin, amlodipine/valsartan/hydrochlorothiazide, amlodipine, moxonidine, acetylsalicylic acid, allopurinol, and esomeprazole.

The patient required hospitalization. ALT was 190 and 239 U/L, and CK was 3,157; 4,498, and 6,704 U/L. Rhabdomyolysis was considered a possible statin-associated adverse reaction. The rosuvastatin–ezetimibe combination was discontinued, and treatment was changed to ezetimibe monotherapy (10 mg/day). Post-withdrawal laboratory results were not available in the reviewed record. In the setting of rhabdomyolysis, muscle injury may have contributed to the aminotransferase elevations; the available findings did not establish a separate drug-induced liver injury.

Available pharmacogenetic results identified the following variants and genotypes: *ABCG2* c.421C**A**, *CYP3A5* *3/*3, and *SLCO1B1* c.388AA, c.463C**A**, c.1929AA, and c.521T**C**, and *CYP2C19* *1/*17.

### 3.10 Case 10

A 31-year-old man with familial hypercholesterolemia developed liver enzyme elevations during treatment with a fixed-dose combination of rosuvastatin (20 mg/day) and ezetimibe (10 mg/day). No additional comorbidities or concomitant medications were recorded in the available documentation. After 2 months of therapy, laboratory findings showed AST 49 U/L, ALT 133 U/L, and GGT 55 U/L. Renal function was preserved, creatinine 84 μmol/L and eGFR 107 mL/min/1.73 m^2^. The rosuvastatin dose was reduced to 10 mg/day, while ezetimibe was continued at 10 mg/day. Repeat testing after 3 months showed improvement, although aminotransferase elevations persisted (AST 42 U/L, ALT 76 U/L, and GGT 46 U/L). The rosuvastatin dose was subsequently reduced to 5 mg/day, while ezetimibe was continued at 10 mg/day. Baseline liver enzyme values, CK measurements, and laboratory results after the second dose reduction were not available in the reviewed records.

Available pharmacogenetic results identified *ABCG2* c.421C**A**, *SLCO1B1* c.521TT; *CYP2C9* *1/*1, *CYP3A4* *1/*1, and *CYP3A5* *3/*3.

### 3.11 Case 11

A 48-year-old man with dyslipidemia, non-obstructive coronary artery disease, hypertension, and obesity (body mass index [BMI] 35.1 kg/m^2^) developed elevated CK and liver enzymes approximately 9 months after starting rosuvastatin (10 mg/day) and ezetimibe (10 mg/day). Concomitant medications included perindopril and acetylsalicylic acid. Approximately 10 months before starting this treatment, CK of 1,700 U/L had been recorded during an episode of chest pain. CK levels immediately before treatment were not available. During combination therapy, laboratory findings were CK 1,244 U/L, AST 62 U/L, and ALT 69 U/L. The patient reported no muscle pain or weakness. An immunological work-up showed no evidence of immune-mediated myopathy. Rosuvastatin was discontinued, while ezetimibe (10 mg/day) was continued. One month later, CK decreased to 738 U/L. After approximately 3 months of ezetimibe monotherapy, CK increased to 5,466 U/L, with AST 148 U/L and ALT 83 U/L. Discontinuation of ezetimibe was then recommended. Follow-up laboratory results after withdrawal were not available.

Pharmacogenetic testing identified *ABCG2* c.421C**A** and *SLCO1B1* c.521T**C**. Given the marked CK elevation, muscle injury may have contributed to the elevated aminotransferases. The available data were insufficient to establish a separate liver injury or attribute the abnormalities to ezetimibe.

## 4 Discussion

Statins and ezetimibe are generally considered safe and effective and are frequently used together to improve lipid control in both primary and secondary cardiovascular prevention (Kei et al., 2016; Ouchi et al., 2019; Lloyd-Jones et al., 2022; Sydhom et al., 2024). Nevertheless, adverse reactions may limit their use in a subset of patients, particularly during intensive lipid-lowering therapy or in the presence of additional clinical and pharmacogenetic risk factors (Guyton et al., 2014; Rosenson et al., 2017; Licata et al., 2019; Preiss et al., 2020).

Statin-associated liver injury is most commonly characterized by elevations in serum transaminases, most often with a hepatocellular pattern, although cholestatic and mixed patterns have also been reported (Björnsson et al., 2012; Newman et al., 2019; Averbukh et al., 2022; Fontana et al., 2023). Mild and transient liver enzyme elevations occur in up to 3% of statin-treated patients, whereas clinically significant liver injury and acute liver failure are rare (Russo et al., 2014; Ramkumar et al., 2016; Newman et al., 2019; Reith et al., 2026). Because aminotransferase elevations may reflect underlying liver disease, concomitant medications, or other clinical conditions, alternative causes should be considered before attributing the abnormalities to statin therapy. In many patients, mild elevations are self-limited and may resolve despite continued treatment, but persistent elevations, positive rechallenge, and the need for treatment discontinuation have also been described (Newman et al., 2019; Reith et al., 2026).

Sporadic cases of liver injury have also been reported for ezetimibe, either as monotherapy or in combination with statins, including cholestatic hepatitis and autoimmune-like liver injury (Van Heyningen, 2005; Stolk et al., 2006; Liu et al., 2007; Castellote et al., 2008; Han et al., 2026). International safety reviews have supported a causal association between ezetimibe and drug-induced liver injury in rare cases (Health Canada, 2024; Han et al., 2026). Randomized clinical trials are usually not powered to detect these events, and much of the currently available evidence relies on pharmacovigilance data, case reports, and mechanistic studies (Björnsson, 2017; Bozina et al., 2018; Meurer and Cohen, 2020; Onfiani et al., 2021; Merćep et al., 2022; Zhou et al., 2025).

In the presented case series, 11 patients had liver enzyme elevations observed during combined statin–ezetimibe therapy. Six also had muscle symptoms and/or elevated CK, including two patients with rhabdomyolysis. In Case 8, rhabdomyolysis was accompanied by acute kidney injury requiring hemodialysis. Laboratory findings improved after treatment modification in several patients, but abnormalities persisted in others. In Cases 1 and 11, abnormalities persisted or increased during subsequent ezetimibe monotherapy, although this did not establish ezetimibe as the cause. Follow-up laboratory results were not available for all patients.

All patients carried the *ABCG2* c.421A allele, as required by the inclusion criteria; eight had the CA genotype and three had the AA genotype. This distribution reflects patient selection and cannot be used to assess an association with liver enzyme elevations.

Several patients also carried variants in other genes involved in drug transport and metabolism. *SLCO1B1* c.521TC was present in five of the 11 cases. Among the eight patients with available results, seven carried the *ABCC2* c.1249A allele and five carried *UGT1A1* *28. Cases 2 and 4 shared *SLCO1B1* c.521TC, *ABCC2* c.1249GA, *ABCB1* c.1236TT/c.2677TT/c.3435TT, and *UGT1A1* *1/*28. Case 8 carried *SLCO1B1* c.521TC and *ABCC2* c.1249GA together with *UGT1A1* *28/*28 and CYP2C9 *1/*3 (Supplementary Table S1). These combinations are of interest, but the available data do not establish their combined effect on drug disposition or their contribution to the clinical findings.

The five comparator patients had the *ABCG2* c.421CA genotype and normal liver enzyme levels documented during combination therapy. Additional variants were also present in this group. C1 carried *SLCO1B1* c.521TC, while C5 carried *ABCC2* c.1249GA and c.-24CT together with *CYP3A4* *22 and had normal liver enzyme levels during atorvastatin 80 mg/day and ezetimibe 10 mg/day (Supplementary Table S3). *NAT2* variant diplotypes were also present in all five comparators. Thus, combinations of variants were documented in patients both with and without liver enzyme elevations. Although *SLCO1B1* c.521TC was numerically more frequent among cases, the small groups and differences in treatment and follow-up limit interpretation. *ABCC2* and *UGT1A1* results were available for only one comparator, preventing comparison of these findings across the two groups.

Several cases had additional factors that complicated interpretation, including pre-existing liver disease, renal impairment, polypharmacy, and muscle injury (Supplementary Table S2). In patients with marked CK elevations, muscle injury may have contributed to the increased AST and ALT levels. The pharmacogenetic findings therefore need to be considered alongside the treatment history and clinical course of each patient. They provide a basis for discussing possible DDGIs, but do not establish that these interactions caused the observed abnormalities.

### 4.1 ABCG2 transporter and pharmacogenetic susceptibility to statin and/or ezetimibe adverse effects

The *ABCG2* gene encodes the efflux transporter BCRP (ABCG2). It is widely expressed at the apical membranes of key epithelial barriers, including enterocytes, hepatocytes, renal proximal tubular cells and the blood–brain barrier (Uchida et al., 2011; Burt et al., 2016; Cheung et al., 2019; Harwood et al., 2019; Kukal et al., 2021). Through the efflux of a broad range of endogenous and exogenous substrates, ABCG2 influences drug absorption, distribution, and excretion and may thereby affect systemic exposure, therapeutic efficacy, and the risk of ADRs (Heyes et al., 2018; Hira and Terada, 2018; Megías-Vericat et al., 2021; Cooper-DeHoff et al., 2022; Merćep et al., 2022; Choi et al., 2025). Clinically relevant ABCG2 substrates include numerous drugs from different therapeutic classes: hypolipidemics (Deng et al., 2021), anticancer drugs (De Vries et al., 2007; Agarwal and Elmquist, 2012; Robey et al., 2018; Traxl et al., 2019; Orlando and Liao, 2020; Sharma et al., 2023), antibiotics, antiretrovirals (Polgar et al., 2008; Mo and Zhang, 2012), and antiepileptics (Luna-Tortós et al., 2008; Nakanishi et al., 2013; Römermann et al., 2015; Bora et al., 2026). Additional substrates include calcium channel blockers (Shukla et al., 2006), sulfasalazine (Safar et al., 2019), proton pump inhibitors (PPIs, e.g., omeprazole, pantoprazole), anticoagulants (apixaban and rivaroxaban), and cyclosporine (Mao and Unadkat, 2015; Heyes et al., 2018; Hira and Terada, 2018; Safar et al., 2019). Various natural products have also been identified as ABCG2 substrates (Sesink et al., 2005; Enokizono et al., 2007; Robey et al., 2011).

ABCG2 is also susceptible to inhibition by numerous drugs, creating the potential for clinically relevant DDIs (Mao and Unadkat, 2015; Toyoda et al., 2019). Co-administration of inhibitors such capmatinib (Grande et al., 2021), febuxostat (Lehtisalo et al., 2020), fenebrutinib (Jones et al., 2020) and fostamatinib (Martin et al., 2016) affects the pharmacokinetics of orally administered ABCG2 substrate drugs. Similarly, some natural products and food additives may interfere with intestinal ABCG2 (Pick et al., 2011; Tan et al., 2013; Sjöstedt et al., 2017). Importantly for the present clinical context, several commonly used drugs, including apixaban, atorvastatin, dabigatran etexilate, fenofibrate, fluvastatin, indomethacin, loratadine, lovastatin, nifedipine, omeprazole, simvastatin, sulfasalazine, tacrolimus, ticagrelor, and vitamin D have been identified as ABCG2 inhibitors *in vitro* (Deng et al., 2023). These observations provide a potential pharmacokinetic basis for further evaluation of ABCG2-mediated interactions in patients receiving multiple medications, but inhibition *in vitro* does not by itself establish a clinically relevant interaction at therapeutic doses.

ABCG2 also participates in the transport of endogenous compounds, including uric acid (Woodward et al., 2009), steroids (Järvinen et al., 2018; 2020), the phospholipid sphingosine-1 phosphate, vitamins, hem, and its precursor protoporphyrin IX (Jonker et al., 2002; Krishnamurthy et al., 2007; Blazquez et al., 2012; Ganguly et al., 2021), emphasizing the physiological relevance of this transporter, beyond drug disposition.

The *ABCG2* c.421C>A (rs2231142, p. Gln141Lys) variant is among the most extensively studied *ABCG2* polymorphisms in this gene. It is associated with reduced transporter expression and function, resulting in decreased ATP-dependent efflux of multiple substrates (Furukawa et al., 2009; Giacomini et al., 2013; Kondo et al., 2004; Fohner et al. 2017). Depending on the substrate, this may increase systemic drug exposure and contribute to susceptibility to adverse drug reactions (Abd and Jacobson, 2011).

Considering the lipid-lowering therapy, the pharmacokinetic relevance of this variant has been most clearly demonstrated for rosuvastatin, where variant allele carriers show approximately 1.5–2-fold higher systemic exposure compared with non-carriers (Keskitalo et al., 2009; Tomlinson et al., 2010; Birmingham et al., 2015). The c.421C>A variant has also been associated with an increased risk of statin-related toxicity, particularly muscular adverse reactions, and in some reports also hepatic adverse reactions, likely reflecting higher systemic exposure observed in variant allele carriers (Merćep et al., 2022; Alqasrawi et al., 2025b; 2025a; Lee et al., 2025). A recent meta-analysis linked *ABCG2* rs2231142 to overall statin toxicity, with odds ratios of 2.6 for muscular and 2.7 for hepatic toxicity (Lee et al., 2025). Associations with variability in statin efficacy have also been reported (Liu et al., 2024).

Evidence concerning rosuvastatin–ezetimibe combination therapy is particularly relevant to the present review. In a prospective observational study, Alqasrawi et al. reported an approximately threefold higher risk of liver enzyme elevation among rosuvastatin-treated carriers of rs2231142, with a particularly pronounced association among East Asian patients (Alqasrawi et al., 2025a). Rosuvastatin use in combination with ezetimibe was also associated with higher risks of muscle symptoms and liver enzyme elevation (Alqasrawi et al., 2025a). These findings support further investigation of treatment and genotype effects, but do not establish that a transporter-mediated interaction caused the observed abnormalities. The biological plausibility of this association is supported by the involvement of ABCG2 in both statin disposition and the enterohepatic recirculation of ezetimibe and its pharmacologically active glucuronide metabolite (Figure 2) (Kosoglou et al., 2005; Oswald et al., 2006; De Waart et al., 2009). Reduced ABCG2 activity may therefore alter hepatic and intestinal handling of both drugs and their metabolites, although the resulting effects on systemic and intrahepatic exposure also depend on other transporters and metabolic pathways. In this context, our findings are consistent with the hypothesis that, in selected patients, liver enzyme elevations observed during statin–ezetimibe therapy may reflect the combined influence of drug exposure, transporter-mediated drug interactions, and pharmacogenetic variability, rather than the effect of a single isolated factor. However, the present case series cannot establish such a mechanism, and these observations should therefore be considered hypothesis-generating.

**FIGURE 2:**
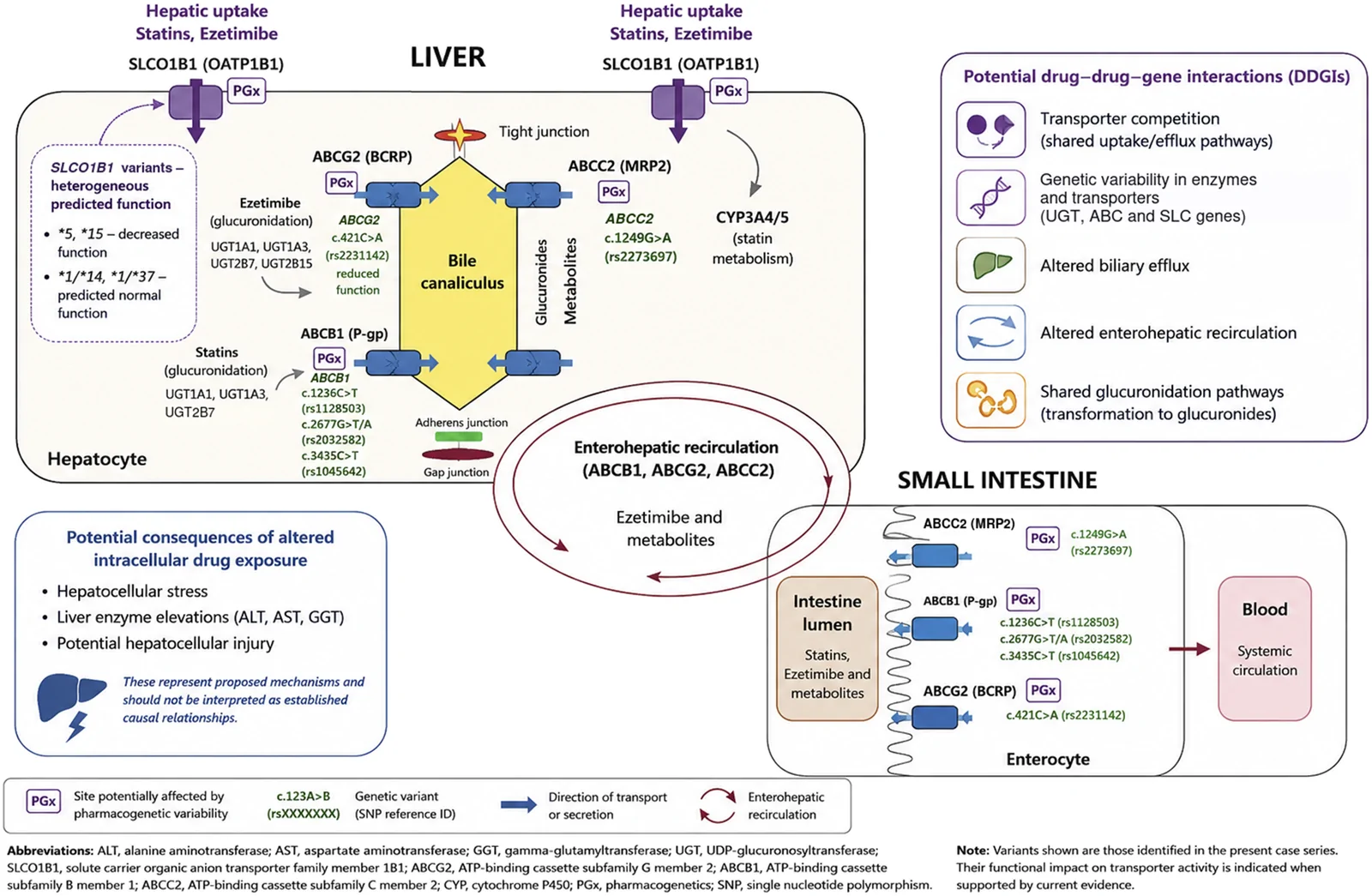
Schematic representation of transporter-mediated disposition of statins and ezetimibe and pharmacogenetic variants relevant to the present case series. Hepatic uptake and biliary and intestinal efflux involve OATP1B1 (SLCO1B1), BCRP (ABCG2), MRP2 (ABCC2), and P-glycoprotein (ABCB1), while ezetimibe undergoes extensive glucuronidation and enterohepatic recycling. The pharmacogenetic variants highlighted in the figure were identified in the present series and may contribute to interindividual variability in drug disposition. The proposed effects on intracellular exposure and liver enzyme elevations represent potential mechanisms and should not be interpreted as established causal relationships. Abbreviations: ALT, alanine aminotransferase; AST, aspartate aminotransferase; GGT, gamma-glutamyltransferase; UGT, UDP-glucuronosyltransferase; SLCO1B1, solute carrier organic anion transporter family member 1B1; ABCG2, ATP-binding cassette subfamily G member 2; ABCB1, ATP-binding cassette subfamily B member 1; ABCC2, ATP-binding cassette subfamily C member 2; CYP, cytochrome P450; PGx, pharmacogenetics; SNP, single nucleotide polymorphism. Note: Variants shown are those identified in the present case series. Their functional impact on transporter activity is indicated when supported by current evidence.

Beyond conventional DDIs, the role of *ABCG2* variants in drug–gene and drug–drug–gene interactions has also been increasingly recognized (Malki and Pearson, 2020; Bruckmueller and Cascorbi, 2021), further supporting the potential clinical relevance of ABCG2 variability in the setting of polypharmacy.

The frequency of the *ABCG2* c.421A allele varies between populations. The global prevalence of the *ABCG2* c.421C>A (rs2231142) variant is approximately 11% (gnomAD, 2026), with substantially higher frequencies in Asian populations (22%–32%), compared with Europeans (7%–14%) and Africans (∼2%) (Furukawa et al., 2009; Woodward et al., 2009; Basseville et al., 2012; Božina et al., 2022; All of Us, 2026). In the Croatian population, the A-allele frequency was reported as 9.7%, with CA and AA genotype frequencies of 17.8% and 0.8%, respectively (Božina et al., 2022). These differences are relevant when interpreting pharmacogenetic studies conducted in different populations.

The importance of ABCG2 in drug disposition is also reflected in regulatory guidance. The US Food and Drug Administration (FDA) and European Medicines Agency (EMA) guidance documents have included evaluation of ABCG2-mediated interactions during drug development, including *in vitro* assessment and, when warranted, clinical DDI studies (European Medicines Agency, 2012; U.S. Food and Drug Administration, 2017).

### 4.2 Relevance of ABCB1, ABCC2 and SLCO1B1 transporters for statin–ezetimibe therapy

Transporters other than ABCG2 might also be relevant to the pharmacokinetic context of the present case series, as ezetimibe undergoes extensive intestinal and hepatic processing, including glucuronidation and enterohepatic recirculation, involving multiple membrane transporters (Oswald et al., 2006; Oswald et al., 2011; De Waart et al., 2009).

Among these transporters, P-glycoprotein, encoded by *ABCB1* gene, and multidrug resistance protein 2 (MRP2), encoded by *ABCC2,* are particularly relevant because they participate in intestinal efflux and hepatobiliary excretion of a wide range of xenobiotics, including lipid-lowering agents and their metabolites. Common *ABCB1* polymorphisms, especially c.1236C>T, c.2677G>T/A and c.3435C>T, have been associated with different transporter expression or activity, although their functional and pharmacokinetic effects vary across substrates and populations (Hoffmeyer et al., 2000; Kajinami et al., 2004; Keskitalo et al., 2008; Peters et al., 2010; Kitzmiller et al., 2013; Su et al., 2015). Associations with statin pharmacokinetics and clinical response are also inconsistent, although some studies have reported increased statin exposure or altered lipid-lowering response in carriers of selected *ABCB1* variants or haplotypes, while others have found no significant association (Zhou et al., 2013; Lalatović et al., 2023; Keskitalo et al., 2008). The effects of *ABCB1* haplotypes may also differ between the acid and lactone forms of statins. In the “positive” studies, carriers of selected *ABCB1* genotypes (*ABCB1* 1236TT, 2677 non-G or 3435TT) demonstrated higher systemic exposure to statins than non-carriers (Zhou et al., 2013). It has been also suggested that the *ABCB1* TTT/TTT haplotype is associated with around 25% longer terminal half-life of atorvastatin, as compared to CGC/CGC haplotype, and that acid and lactone form of atorvastatin may differ in susceptibility to genetic variants (Keskitalo et al., 2008). The *ABCB1* G2677A/T variant allele carriers treated with atorvastatin experienced greater lipid reductions than the GG (wild-type) subjects (Tian et al., 2025). These findings suggest a possible contribution to treatment variability, but do not justify classifying all three common *ABCB1* polymorphisms as reduced-function variants. From a mechanistic perspective, reduced intestinal ABCB1 activity may prolong intestinal residence time of substrate drugs, increase the absorption of a substrate drug, but the direction and magnitude of a genotype effect cannot be assumed to be the same for all statins.

These polymorphisms are common in European populations, with reported minor allele frequencies of approximately 40%–50%, although their functional effects are variable and context-dependent. In a Croatian reference population, the variant allele frequencies of c.1236C>T (rs1128503) and c.3435C>T (rs1045642) were 57.9% and 48.8%, respectively, comparable to frequencies of 56.6% and 46.1% reported in European gnomAD controls (Celinšćak et al., 2022). In the present series, *ABCB1* variants were frequently observed, particularly c.1236C>T, c.2677G>T/A, and c.3435C>T. Therefore, their frequent occurrence in the present series should not be interpreted as evidence of impaired P-glycoprotein function, but rather as part of the broader pharmacogenetic background potentially influencing interindividual variability in drug disposition.

ABCC2/MRP2 is also highly relevant in this context because it is localized on the canalicular membrane of hepatocytes and plays a central role in hepatobiliary excretion. Several statins are substrates of MRP2, while ezetimibe glucuronide is also transported through this pathway as a part of its enterohepatic recycling (De Waart et al., 2009; Bruhn and Cascorbi, 2014) (Figure 2). The *ABCC2* variants, including c.-24C>T (rs717620) and c.1249G>A (rs 2273697), have been associated with differences in transporter expression or activity, suggesting that genetic variability in MRP2 may influence the disposition of its substrates (Božina et al., 2017; Guan et al., 2019).

Recent studies suggest that genetic variability in these transporters may contribute to interindividual differences in ezetimibe pharmacokinetics (Mauriello et al., 2023; González-Iglesias et al., 2024). For example, Gonzalez-Iglesias et al. reported a trend towards higher ezetimibe C_max_ values in carriers of the *ABCC2* c.1249 G>A (rs2273697) and *ABCB1* c.2677G>T/A (rs2032582) variants. Although these observations do not establish a direct functional or clinical effect, they support the potential relevance of transporter variability to ezetimibe pharmacokinetics, but their functional basis and clinical relevance require further investigation.

In the present series, seven of the eight patients with available *ABCC2* results carried the *ABCC2* c.1249G>A (rs2273697) variant, resulting in the Val417Ile substitution. This is a relatively common variant, with a reported A-allele frequency of approximately 20% in European populations (González-Iglesias et al., 2024). Its recurrent presence in our patients is therefore of potential interest in the context of MRP2-mediated biliary efflux, but cannot be interpreted as enrichment or as evidence of impaired MRP2 function. However, evidence directly linking this variant to liver enzyme elevations during statin–ezetimibe therapy is lacking. Associations reported with hepatotoxicity during treatment with other drugs provide mechanistic context but cannot be directly extrapolated to the present clinical setting.

Hepatic uptake is equally important for the disposition of statins and ezetimibe. Transporter OATP1B1, encoded by the *SLCO1B1* gene, mediates the hepatocellular uptake of several statins and also participates in ezetimibe disposition (Oswald et al., 2008; Kalliokoski and Niemi, 2009; Božina et al., 2022; Park et al., 2022). Genetic variation in this transporter may therefore contribute to interindividual differences in systemic drug exposure and susceptibility to ADRs (Hagenbuch et al., 2025).

For statins, the best-established pharmacogenetic association is the reduced-function *SLCO1B1* c.521T>C (p.Val174Ala, rs4149056) variant. This variant is associated with reduced hepatic uptake, increased systemic exposure, and statin-associated myotoxicity, particularly with simvastatin treatment (Link, 2008; Kee et al., 2020; Turongkaravee et al., 2021; Cooper-DeHoff et al., 2022). Its clinical relevance was first demonstrated in a genome-wide association study (GWAS) that identified *SLCO1B1* c.521T>C as a major genetic determinant of statin-induced myopathy (Link, 2008). These findings are confirmed in multiple studies and incorporated into clinical pharmacogenetic guidelines (Cooper-DeHoff et al., 2022; Wolthuis et al., 2025).

The relationship between OATP1B1 function and liver injury, however, is different from that observed for myotoxicity. Reduced hepatic uptake variants may increase plasma concentrations without increasing hepatocellular statin exposure to the same extent. In contrast, enhanced-function *SLCO1B1* haplotypes, such as *14 and *20 (Mykkänen et al., 2022), may increase hepatic uptake and could theoretically increase hepatocellular exposure, especially when biliary elimination is impaired (Neuvonen et al., 2021; Ramsey et al., 2023). The contribution of OATP1B1 also differs among individual statins according to their transporter affinity, which may partly explain differences in clinically observed statin gene–drug interactions (Kiander et al., 2025).

OATP1B1 may also be relevant to ezetimibe disposition, as both ezetimibe and ezetimibe glucuronide are OATP1B1 substrates. *In vivo*, the SLCO1B1*1B allele has been associated with lower ezetimibe exposure and altered handling of its glucuronide metabolite, further supporting a role of OATP1B1 in ezetimibe disposition (Oswald et al., 2008; Lee and Ho, 2017). However, these pharmacokinetic observations do not establish an association with liver injury.

In the present series, five patients had the *SLCO1B1* c.521TC genotype and six had c.521TT (Supplementary Table S1). The available variant results did not allow unambiguous assignment of *SLCO1B1* diplotypes in all patients. Although *14 is classified as an increased-function allele, the *1/*14 diplotype is associated with a predicted normal-function phenotype. In European populations, the decreased-function *SLCO1B1* *5 and *15 alleles have been reported at frequencies of approximately 2% and 15%, respectively (Ramsey et al., 2023). The *SLCO1B1* c.521TC genotype was also present in one comparator with documented normal liver enzyme levels during combination therapy (Supplementary Table S3). Nevertheless, the interplay between OATP1B1-mediated hepatic uptake and biliary or intestinal efflux through ABCG2, ABCB1, and ABCC2 could theoretically modify intrahepatic drug exposure (Tátrai et al., 2022). Taken together, these observations highlight the potential importance of the balance between hepatic uptake and biliary/intestinal efflux, rather than systemic drug exposure alone. This may be particularly relevant for ezetimibe, whose pharmacokinetics are strongly influenced by glucuronidation and enterohepatic recycling rather than by a simple one-pass elimination model.

### 4.3 Role of CYPs and UGTs polymorphisms as an additional layer of variability

While transporter variability represents the most recurrent pharmacogenetic finding in the present patient series, metabolic enzymes may provide an additional layer of variability in drug disposition and drug response. Compared with transporter genes, the distribution of metabolic enzyme variants was less consistent across our patients, suggesting that their potential contribution should be considered within the broader pharmacokinetic context rather than as an independent explanation for the observed clinical phenotypes (Supplementary Table S1). Statins and ezetimibe differ substantially in their metabolism. Atorvastatin, a relatively lipophilic statin, is extensively metabolized in the intestine and liver, mainly through CYP3A4 and CYP3A5, whereas rosuvastatin, a more hydrophilic statin, undergoes only limited CYP-mediated metabolism, primarily through CYP2C9 and, to a lesser extent, CYP2C19 (Hirota et al., 2020; McIver and Siddique, 2026). In contrast, for ezetimibe, CYP-mediated metabolism is not the dominant metabolic route; instead, phase II glucuronidation represents its major metabolic pathway. The potential relevance of CYP3A4/5 variability in the present series is therefore greatest in patients treated with atorvastatin. Functional variation in *CYP3A4*, particularly the reduced-function *CYP3A4* *22 allele, may influence atorvastatin exposure and could contribute to interindividual variability in treatment response and tolerability (Park et al., 2008; 2022; Mykkänen et al., 2024).

In our series, all patients were CYP3A5 non-expressers (*3/*3 genotype; associated with absent CYP3A5 expression). Given the high prevalence of CYP3A5 *3/*3 in European populations, its presence across the series cannot be considered an unusual finding or interpreted as evidence of an association with the observed liver enzyme abnormalities. The CYP3A4*22 allele was identified in one of the ten cases with available results and in one of the four comparators with available results (Supplementary Tables S1, S3). Nevertheless, *CYP3A4*/*5* genotype may form part of the overall pharmacokinetic background, particularly in patients receiving atorvastatin together with other drugs that engage CYP3A activity.

For rosuvastatin, the role of CYP-mediated metabolism is considerably smaller, making transporter-mediated disposition more relevant to its pharmacokinetic variability. CYP polymorphisms alone are therefore unlikely to account for the clinical findings observed in rosuvastatin-treated patients, although metabolic and transporter variability may act jointly to modify drug disposition. For ezetimibe, the more relevant metabolic enzymes are UGT1A1, UGT1A3, UGT2B7 and, to a lesser extent, UGT2B15, which participate in the formation of ezetimibe glucuronide (Prueksaritanont et al., 2002; Bae et al., 2011; Stormo et al., 2013; Schirris et al., 2015; Hanke et al., 2021; Shi and Han, 2025). Polymorphisms within the *UGT1A* locus may influence enzyme expression and activity and thereby contribute to interindividual variability in glucuronidation (Bae et al., 2011; Stormo et al., 2013; Schirris et al., 2015; Hanke et al., 2021; Mykkänen et al., 2024). This may be relevant because ezetimibe and its glucuronide undergo extensive enterohepatic recirculation, while statins and ezetimibe also share several glucuronidation pathways: atorvastatin is glucuronidated primarily by UGT1A3, UGT1A1 and UGT2B7, rosuvastatin mainly by UGT1A1 and UGT1A3, and ezetimibe by UGT1A1, UGT1A3, UGT2B7 and UGT2B15 (Prueksaritanont et al., 2002; Schirris et al., 2015; Hanke et al., 2021; Shi and Han, 2025). However, current evidence supporting a clinically relevant pharmacogenetic effect of *UGT* variants in ezetimibe therapy remains limited, and no clinically actionable pharmacogenetic recommendations are currently available. *UGT* variability should therefore be regarded as a biologically plausible component of the broader pharmacokinetic framework rather than as an established determinant of liver enzyme elevations during statin–ezetimibe therapy. Among the eight cases with available *UGT1A1* results, four had *1/*28 and one had *28/*28. The homozygous genotype was present in Case 8, together with several transporter and CYP variants. *UGT2B7* c.-161C>T also varied among the tested cases (Supplementary Table S1). These findings are relevant to the description of their pharmacogenetic profiles, but do not establish altered ezetimibe metabolism in individual patients. *UGT* results were available for only one comparator, limiting comparison between the groups. Evidence linking these variants to liver enzyme elevations during statin–ezetimibe therapy remains insufficient to support genotype-based prediction of this outcome.

A possible role of *NAT2* variants has also emerged from a pharmacokinetic study of rosuvastatin–ezetimibe therapy, in which intermediate and poor acetylators were associated with higher rosuvastatin exposure than rapid acetylator phenotypes (González-Iglesias et al., 2024). However, neither rosuvastatin nor ezetimibe is an established substrate of NAT2, and the mechanism behind this finding remains unclear. In our series, *NAT2* results were available for eight cases and all five comparator patients. Variant *NAT2* diplotypes were present in both groups, with considerable overlap between cases and comparators (Supplementary Tables S1, S3). Given the small number of patients, these findings do not allow any conclusion on the contribution of NAT2 to the observed liver enzyme elevations.

### 4.4 Drug–drug–gene interactions

Atorvastatin, rosuvastatin, and ezetimibe share several polymorphic metabolic pathways, including UGT-mediated glucuronidation, ABC efflux transporters, and SLC uptake transporters. Genetic variation and concomitant medications may affect these pathways, providing a biologically plausible basis for both drug–drug interactions (DDIs) and drug–drug–gene interactions (DDGIs) (Tod et al., 2020). However, the involvement of two drugs in a shared pathway does not itself establish a clinically relevant interaction, and the presence of a pharmacogenetic variant does not necessarily increase its magnitude.

Physiologically based pharmacokinetic (PBPK) models provide a means of examining the contribution of individual pathways to drug exposure and interactions. For statins, these models illustrate the complex contribution of intestinal absorption, hepatic uptake and metabolism, and biliary or renal elimination (Yoshikado et al., 2017; Yang et al., 2020). For atorvastatin, such models identify OATP-mediated hepatic uptake and CYP3A metabolism as major determinants of disposition, while more recent approaches have incorporated genetic polymorphisms and DDIs, illustrating how multiple factors may jointly determine drug exposure (Yang et al., 2020). For rosuvastatin, a recent PBPK model incorporated genetic polymorphisms and drug–drug interactions (Balhara et al., 2025).

For atorvastatin, numerous clinically relevant DDIs have been described particularly with inhibitors of CYP3A4 and/or drug transporters (Hirota and Ieiri, 2015; Hirota et al., 2020). Importantly, coadministration of atorvastatin (40 mg/day) and ezetimibe (10 mg/day) for 7 days increased total exposure to atorvastatin by 21% and 2-hydroxyatorvastatin exposure by 8%, indicating that even the statin–ezetimibe combination itself may modestly alter atorvastatin disposition (Park et al., 2017). Similarly, coadministration of atorvastatin with sacubitril/valsartan therapy increased the exposure to atorvastatin and its metabolites by 38% and 24%, respectively (Ayalasomayajula et al., 2017). Although these interaction magnitudes are modest compared with those observed with strong CYP3A inhibitors (Huang et al., 2017), their clinical relevance may vary according to the patient’s pharmacogenetic background, concomitant medications, and other factors affecting drug disposition. For rosuvastatin, transporter-mediated interactions are particularly relevant because the drug undergoes limited CYP-mediated metabolism (Hirota et al., 2020). Inhibition of BCRP/ABCG2 and OATP1B1/OATP1B3 can substantially increase rosuvastatin exposure, as demonstrated by interactions with potent inhibitors such as cyclosporine and darolutamide (Simonson, 2004; Zurth et al., 2019). The magnitude of such interactions may depend not only on the inhibitory properties of a co-medication but also on the patient’s transporter genotype and other interacting factors. The interaction between rosuvastatin and ezetimibe deserves particular attention. A recent population pharmacokinetic/pharmacodynamic (PK/PD) model incorporating enterohepatic recirculation concluded that the overall pharmacokinetic interaction between rosuvastatin and total ezetimibe was not clinically significant at the population level (An and Shin, 2025). However, this does not exclude clinically meaningful interactions in selected patients. In a pharmacogenetic study of 119 healthy volunteers participating in rosuvastatin-alone and rosuvastatin-plus-ezetimibe pharmacokinetic trials, the combined regimen was associated with a significant prolongation of rosuvastatin half-life compared with rosuvastatin alone (González-Iglesias et al., 2024). Decreased- and poor-function ABCG2 phenotypes were associated with significantly higher peak and total exposure to rosuvastatin, while reduced-function SLCO1B1 phenotypes also contributed to increased drug levels. The same study identified differences according to NAT2 acetylator status, further illustrating that the pharmacokinetics of combination therapy may reflect the contribution of several pathways rather than a single pharmacogene (González-Iglesias et al., 2024).

From the clinical perspective, polypharmacy is common in patients receiving intensive lipid-lowering therapy, increasing the probability of clinically relevant DDIs (Očovská et al., 2023). This is particularly relevant to the present series, in which several patients received concomitant medications with the potential to affect pathways involved in statin exposure. Ticagrelor, for example, is commonly used together with rosuvastatin after atherothrombotic events, and cases of rhabdomyolysis have been reported during concomitant therapy (Vrkić Kirhmajer et al., 2018). More recent experimental data have shown that ticagrelor inhibits BCRP and OATP transporters, providing a mechanistic explanation for increased rosuvastatin exposure (Lehtisalo et al., 2024). Similarly, proton pump inhibitors and loop diuretics have been associated with modest changes in atorvastatin exposure, with evidence that the magnitude of such interactions may vary according to metabolizer status (Turner et al., 2020a). Case reports have also suggested that pantoprazole–atorvastatin interactions may become clinically relevant in genetically susceptible individuals (Marusic et al., 2012). Such observations require cautious interpretation because concomitant disease, other medications, and treatment differences may also contribute.

Taken together, these observations illustrate why average effects derived from conventional pharmacokinetic interaction studies may not fully capture interindividual variability. At the population level, the pharmacokinetic interaction between rosuvastatin and ezetimibe appears modest (Wang et al., 2023; An and Shin, 2025), but the net effect in an individual patient may depend on the combined influence of transporter function, metabolic capacity, concomitant medications, and other clinical factors.

In the present series, several patients received medications relevant to these pathways (Supplementary Table S2). Case 6, for example, received ticagrelor with rosuvastatin–ezetimibe and carried the *ABCG2* c.421CA genotype. The documented rosuvastatin–ticagrelor interaction is therefore relevant to the assessment of this patient, but the additional contribution of genotype cannot be determined. Concomitant medications and pharmacogenetic variants were also present in the comparator patients with documented normal liver enzyme levels during combination therapy (Supplementary Table S3).

In the present case series, this framework offers a biologically plausible context for interpreting the observed liver enzyme elevations, but the absence of pharmacokinetic measurements and a formal assessment of genotype-dependent interactions prevents attribution of the observed abnormalities to a specific DDGI.

### 4.5 Clinical implications

Ezetimibe is generally well-tolerated, and the present observations do not alter its established role in lipid-lowering therapy (Kei et al., 2016; Ouchi et al., 2019). However, the present cases illustrate the need to assess liver enzyme elevations during statin–ezetimibe therapy in the context of the complete treatment regimen and the patient’s clinical history.

When abnormalities occur, their timing in relation to treatment initiation, dose escalation, and changes in concomitant medications should be reviewed, together with baseline findings and the course after treatment modification. Assessment should also consider pre-existing liver disease, renal impairment, and other possible causes. In patients with muscle symptoms or marked CK elevations, a muscle contribution to AST and ALT elevations should be considered before interpreting these findings as hepatic injury. Improvement after withdrawal of combination therapy may support a treatment-related event, but does not identify the responsible drug. In several patients in this series, liver enzyme abnormalities occurred during ezetimibe exposure in combination with a statin and improved following treatment modification or discontinuation, whereas abnormalities persisted in others or follow-up was incomplete. These differences underline the importance of documenting the clinical course rather than attributing all abnormalities to a statin class effect. Ezetimibe and concomitant medications should also be considered in the assessment, without assuming that their involvement has been established. Decisions on dose reduction, treatment interruption, or an alternative regimen should reflect the severity and persistence of the abnormalities, associated symptoms, and the patient’s cardiovascular risk.

Pharmacogenetic information may provide additional context when evaluating selected patients with recurrent or otherwise unexplained intolerance to lipid-lowering therapy, particularly when interpreted alongside treatment exposure, concomitant medications, and clinical characteristics. Transporter genes such as *ABCG2* and *SLCO1B1*, and possibly *ABCB1* and *ABCC2*, are of mechanistic interest because of their roles in statin and ezetimibe disposition (Hirota et al., 2020; Cooper-DeHoff et al., 2022). Current Clinical Pharmacogenetics Implementation Consortium (CPIC) recommendations for *SLCO1B1*, *ABCG2*, and *CYP2C9* address statin selection and dosing in relation to statin-associated musculoskeletal symptoms (Cooper-DeHoff et al., 2022). They should not be interpreted as recommendations for predicting liver injury during statin–ezetimibe therapy. The clinical relevance of *ABCB1*, *ABCC2*, and *UGT* variants in this setting remains uncertain, and their presence alone does not justify treatment modification or expanded pharmacogenetic testing. The comparator patients further illustrate this distinction: pharmacogenetic variants were also present in patients with documented normal liver enzyme levels during combination therapy (Supplementary Table S3). Although this small group cannot establish comparative risk, it cautions against using an individual variant or a combination of variants as an explanation for intolerance without supporting clinical evidence. The present findings should therefore be seen as hypothesis-generating and as a rationale for further investigation rather than as a basis for clinical testing recommendations. The practical implication of this series is therefore the value of an individual assessment that brings together treatment history, laboratory findings, concomitant medications, and available pharmacogenetic information. Whether broader pharmacogenetic profiling improves the prediction or management of liver enzyme elevations during statin–ezetimibe therapy remains to be established in larger studies with appropriate comparison groups and consistent clinical assessment.

### 4.6 Strengths and limitations

The strength of this case series lies in the combined description of treatment history, available laboratory follow-up, concomitant medications, and pharmacogenetic findings across several pathways relevant to statin and ezetimibe disposition. These data allowed individual clinical courses and potential contributing factors to be examined, although the extent of documentation varied between patients. Carriage of the *ABCG2* c.421A allele was an inclusion criterion and should not be interpreted as an independent finding supporting an association with liver enzyme elevations.

Several limitations should also be acknowledged. Evidence regarding importance of pharmacogenetic factors for the efficacy and safety of ezetimibe is substantially more limited than that available for statins. Although there is a strong mechanistic rationale, particularly regarding transporter-mediated drug disposition and enterohepatic recirculation, clinically actionable pharmacogenetic guidance for ezetimibe is currently lacking. In this context, the observations from the present small patient series should be viewed as exploratory and hypothesis generating.

Five patients with documented normal liver enzyme findings during combination therapy were included for descriptive comparison. However, the small comparator group, differences in treatment and follow-up, and incomplete laboratory and pharmacogenetic data do not allow reliable assessment of variant enrichment or genotype-associated risk. Selection of cases for carriage of the *ABCG2* c.421A allele further limits interpretation of its association with the observed abnormalities. In addition, because the patients were treated in a real-world clinical setting and received multiple concomitant medications, the relative contributions of statins, ezetimibe, co-medications, and underlying clinical factors cannot be reliably disentangled in individual cases. Other potential non-genetic contributors, such as diet, alcohol consumption, gut microbiota composition, and subclinical liver disease, were not systematically evaluated.

Although the temporal relationship between therapy modification and improvement or normalization of liver enzymes in several patients is compatible with a treatment-related effect, rechallenge was not systematically performed, and in some cases more than one lipid-lowering agent was modified during follow-up. A structured descriptive assessment of potential contributing factors and limitations of causality assessment was performed for each patient (Supplementary Table S2). RUCAM scores were not calculated because the retrospective clinical information required for reliable scoring was not consistently available. Baseline and follow-up laboratory values and precise sampling dates were incomplete in several cases, limiting assessment of the course after treatment modification (Supplementary Table S4). Furthermore, pre-existing liver abnormalities, muscle injury, polypharmacy, and treatment modifications involving more than one lipid-lowering agent would have limited the interpretability of retrospective causality scores. Consequently, attribution of the observed liver enzyme abnormalities to an individual drug or drug combination remains uncertain.

These observations do not establish that DDGIs caused the liver enzyme abnormalities observed in our patients, but they identify a clinically relevant hypothesis that warrants evaluation in larger, controlled studies.

## 5 Conclusion

This series describes liver enzyme elevations during statin–ezetimibe therapy in 11 patients selected for carriage of the *ABCG2* c.421A allele. The clinical course, concomitant treatment, and additional pharmacogenetic findings varied across patients. Five comparator patients carrying the CA genotype had documented normal liver enzyme levels during combination therapy, illustrating that this genotype can be present in patients with and without such abnormalities.

The accompanying narrative review provides a pharmacokinetic rationale for investigating the combined effects of genetic variation and concomitant medications. However, this case series cannot establish the contribution of individual variants or DDGIs to the observed abnormalities. These observations remain hypothesis-generating and warrant further investigation in studies with appropriate comparison groups, standardized clinical assessment, and pharmacokinetic measurements. A better understanding of the interplay between drug transporters, metabolic pathways, concomitant medications, and pharmacogenetic variability may ultimately contribute to more individualized lipid-lowering therapy.

## Data Availability

The datasets presented in this study can be found in online repositories. The names of the repository/repositories and accession number(s) can be found in the article/Supplementary Material.
