# Supplementary material for: Liver enzyme elevations during statin–ezetimibe therapy in ABCG2 c.421C>A variant carriers: a case series and narrative review
Source: Front Pharmacol. 2026 Oct 5;17:1840055. doi: 10.3389/fphar.2026.1840055 (PMC13677550; doi:10.3389/fphar.2026.1840055)
Supplement: Supplementary file 1 [file Table1.docx]

| **Supplementary Table 1.** Detailed pharmacogenetic results for the 11 patients included in the case series. | | | | | | | | | | | | | | | | | | | |
| --- | --- | --- | --- | --- | --- | --- | --- | --- | --- | --- | --- | --- | --- | --- | --- | --- | --- | --- | --- |
| **Case** | ***CYP2C9*** | ***CYP2C19*** | ***CYP3A4*** | | ***CYP3A5*** | ***NAT2*** | ***ABCB1*** | | | ***ABCC2*** | | ***ABCG2*** | | ***SLCO1B1*** | | | | ***UGT1A1*** | ***UGT2B7*** |
|  | **2, *3* | **2, *17* | **1B* | **22* | **3* | **5, *6, *7* | c.1236C>T  ex 12 | c.2677  G>T/A  ex 21 | c.3435C>T  ex 26 | c.-24 C>T ex 1 | c.1249  G>A  ex 10 | c.34  G>A | c.421  C>A | c.388  A>G | c.463  C>A | c.1929  A>C | c.521  T>C | **28* | c.-161 C>T |
| **1** | **1/*1* | ****2/*17*** | **1/*1* | **1/*1* | **3/*3* | ****6/*6*** | CC | GG | CC | CC | G**A** | GG | C**A** | A**G** | C**A** | AA | TT | **1/*****28*** | CC |
| **2** | **1/*1* | ******2****/*****17*** | **1/*1* | **1/*1* | **3/*3* | **4/*****6*** | **TT** | **TT** | **TT** | CC | G**A** | GG | **AA** | AA | CC | AA | T**C** | **1/*****28*** | CC |
| **3** | **1/*****2*** | **1/*****17*** | **1/*1* | **1/*1* | **3/*3* | **4/*****5*** | C**T** | G**T** | C**T** | CC | GG | GG | **AA** | A**G** | CC | AA | TT | **1/*1* | CC |
| **4** | **1/*****2*** | **1/*****17*** | **1/*1* | **1/*1* | **3/*3* | ****6/*6*** | **TT** | **TT** | **TT** | CC | G**A** | GG | C**A** | A**G** | CC | AA | T**C** | **1/*****28*** | C**T** |
| **5** | **1/*1* | **1/*****17*** | **1/*1* | **1/*1* | **3/*3* | **4/*****6*** | C**T** | G**T** | C**T** | CC | G**A** | GG | C**A** | AA | CC | AA | TT | **1/*1* | CC |
| **6** | **1/*1* | **1/*****17*** | **1/*1* | **1/*1* | **3/*3* | **4/*****5*** | **TT** | **TT** | **TT** | CC | G**A** | GG | C**A** | A**G** | CC | AA | TT | **1/*****28*** | C**T** |
| **7** | **1/*1* | **1/*****2*** | **1/*1* | **1/*****22*** | **3/*3* | ****5/*6*** | **TT** | **TT** | C**T** | CC | **AA** | GG | **AA** | A**G** | CC | AA | TT | **1/*1* | CC |
| **8** | **1/*****3*** | **1/*1* | **1/*1* | **1/*1* | **3/*3* | **4/*****5*** | C**T** | G**T** | C**T** | CC | G**A** | GA | C**A** | A**G** | CC | AA | T**C** | ****28/*28*** | **TT** |
| **9** | *NR* | **1/*17* | *NR* | *NR* | **3/*3* | *NR* | NR | NR | NR | NR | NR | NR | C**A** | AA | CA | AA | TC | **NR** | **NR** |
| **10** | **1/*1* | *NR* | *NR* | **1/*1* | **3/*3* | *NR* | NR | NR | NR | NR | NR | NR | C**A** | NR | NR | NR | TT | **NR** | **NR** |
| **11** | **1/*1* | *NR* | *NR* | **1/*1* | **3/*3* | *NR* | NR | NR | NR | NR | NR | NR | C**A** | NR | NR | NR | T**C** | **NR** | **NR** |

Genotypes are reported according to the nomenclature of the respective pharmacogenes. Star (*) allele nomenclature is used where applicable; for transporter variants, genotypes are reported for the individual nucleotide substitutions shown in the table. For individual variants, two identical nucleotides indicate homozygosity, whereas two different nucleotides indicate heterozygosity.

Abbreviations: *ABCB1*, ATP-binding cassette subfamily B member 1; *ABCC2*, ATP-binding cassette subfamily C member 2; *ABCG2*, ATP-binding cassette subfamily G member 2; *CYP*, cytochrome P450; *NAT2*, N-acetyltransferase 2; NR, not reported in the available pharmacogenetic records; this designation does not imply a reference genotype or confirm that testing was not performed; *SLCO1B1*, solute carrier organic anion transporter family member 1B1; *UGT*, UDP-glucuronosyltransferase.
