# Supplementary material for: Liver enzyme elevations during statin–ezetimibe therapy in ABCG2 c.421C>A variant carriers: a case series and narrative review
Source: Front Pharmacol. 2026 Oct 5;17:1840055. doi: 10.3389/fphar.2026.1840055 (PMC13677550; doi:10.3389/fphar.2026.1840055)
Supplement: Supplementary file 2 [file Table2.docx]

**Supplementary Table 2.** Clinical factors, concomitant medications, and descriptive causality assessment in patients with liver enzyme elevations during statin–ezetimibe therapy

| **Patient** | **Relevant comorbidities/clinical factors** | **Concomitant medications** | **Potential contributing factors and limitations of causality assessment** |
| --- | --- | --- | --- |
| 1 | Dyslipidemia; hypertension;  type 2 diabetes mellitus | Esomeprazole, nebivolol, spironolactone, perindopril/indapamide/amlodipine, metformin, meloxicam, dexketoprofen/tramadol, acetaminophen/tramadol, amitriptyline, and alprazolam. | Recurrent liver enzyme elevations during different statin regimens and ezetimibe monotherapy; concomitant CK elevation with a possible muscle contribution to aminotransferase elevations; polypharmacy, including multiple analgesics.  Persistence during ezetimibe monotherapy and later normal liver enzymes on low-dose atorvastatin do not identify a single causal drug. The contribution of individual co-medications or a specific interaction could not be established. |
| 2 | Coronary artery disease;  elevated Lp(a); fatty liver;  long-term lipoprotein apheresis | Nebivolol, acetylsalicylic acid, furosemide, pantoprazole; PCSK9 inhibitor | Pre-existing fatty liver/chronic liver disease; previous liver enzyme elevations during statin treatment; polypharmacy.  Improvement after withdrawal was accompanied by initiation of apheresis. Abnormalities documented years after reintroduction do not establish a positive rechallenge. Underlying liver disease limits attribution to treatment; a specific drug interaction was not demonstrated. |
| 3 | Dyslipidemia;  regular intensive physical exercise | Available records do not indicate relevant concomitant medication | Regular physical exercise and marked CK elevation; possible muscle contribution to AST elevation.  CK was already elevated before ezetimibe was added. Subsequent improvement followed both a statin switch and reduced ezetimibe dosing, preventing attribution to either change. No relevant concomitant medication was documented for assessment of an additional drug interaction. |
| 4 | Familial hypercholesterolemia;  previous MI/CABG/PCI | Amlodipine, bisoprolol, acetylsalicylic acid, pantoprazole, diazepam, alprazolam | Polypharmacy.  Enzymes decreased but remained elevated after treatment changes. The sampling date and exact regimen at follow-up were unclear. Pantoprazole was co-prescribed, but the records do not establish an atorvastatin–pantoprazole interaction as the cause. |
| 5 | Familial hypercholesterolemia; osteoporosis | Rabeprazole, citalopram, alprazolam, melatonin, ibandronic acid, vitamin D | Polypharmacy; concomitant medications as potential contributors. Improvement followed both ezetimibe withdrawal and rosuvastatin dose reduction. Later normalisation followed further treatment changes. These observations do not distinguish the effects of the individual drugs or establish a specific interaction. |
| 6 | Hypertension;  coronary and peripheral arterial disease;  renal artery stenosis;  generalized atherosclerosis | Spironolactone, nebivolol, acetylsalicylic acid, ticagrelor, amlodipine/valsartan | Polypharmacy and substantial cardiovascular comorbidity.  Ticagrelor can increase rosuvastatin exposure; this interaction is supported by clinical pharmacokinetic evidence [1]. Its contribution to liver enzyme elevations in this patient remains unproven. Follow-up laboratory values after rosuvastatin withdrawal were unavailable. |
| 7 | Hypercholesterolemia; statin-associated muscle symptoms (SAMS) | Available records do not indicate relevant concomitant medication | Muscle symptoms during statin treatment; smoking; intermittent/reduced ezetimibe exposure during follow-up.  The earlier muscle symptoms occurred with liver enzymes and CK within reference limits; subsequent ALT elevation was recorded on fluvastatin–ezetimibe. Laboratory follow-up after ezetimibe dose reduction was unavailable. No relevant concomitant medication was documented for assessment of an additional drug interaction. |
| 8 | Dyslipidemia; hypertension; type 2 diabetes mellitus; paroxysmal atrial fibrillation; combined aortic valve disease; previous stroke; prior right nephrectomy for oncocytoma; acute kidney injury requiring hemodialysis during the reported episode | Dapagliflozin/metformin, furosemide, valsartan, alprazolam, bisoprolol, amlodipine/valsartan/hydrochlorothiazide, levothyroxine, apixaban, tramadol/paracetamol | Severe rhabdomyolysis with a possible muscle contribution to aminotransferase elevations; concurrent acute kidney injury requiring hemodialysis; polypharmacy.  Both lipid-lowering drugs were withdrawn during hospitalisation. The timing of laboratory peaks relative to withdrawal was unclear. Concurrent muscle and renal injury limit assessment of a separate hepatic effect; no specific drug interaction was established in this patient. |
| 9 | Dyslipidemia; diabetes mellitus; hypertension; chronic kidney disease (eGFR <15 mL/min/1.73 m²); hemolytic anemia; hyperbilirubinemia; hyperuricemia; generalized atherosclerotic disease | Gliclazide, sitagliptin/metformin, amlodipine/valsartan/hydrochlorothiazide, amlodipine, moxonidine, acetylsalicylic acid, allopurinol, esomeprazole. | Rhabdomyolysis with a possible muscle contribution to aminotransferase elevations; severe renal impairment; hemolytic anemia complicating interpretation of hyperbilirubinemia; polypharmacy.  The available sampling dates did not permit a reliable assessment after statin withdrawal and continuation of ezetimibe. A contribution from muscle injury could not be separated from a hepatic source of aminotransferases. No specific drug interaction was established in this patient. |
| 10 | Familial hypercholesterolemia;  no additional comorbidities documented | No concomitant medications recorded in the available documentation | No specific alternative contributors documented; baseline liver enzyme values and CK measurements were not available in the reviewed records.  Enzymes decreased after rosuvastatin dose reduction while ezetimibe was continued, although aminotransferase elevations persisted. This course is compatible with a treatment-related effect but does not establish causality. No concomitant medication was documented for assessment of an additional drug interaction. |
| 11 | Dyslipidemia;  non-obstructive coronary artery disease;  hypertension;  class II obesity (BMI 35.1 kg/m²);  previous CK elevation during an episode of chest pain | Perindopril, acetylsalicylic acid | Previous CK elevation during chest pain, before combination therapy; possible muscle contribution to aminotransferase elevations. CK immediately before treatment initiation was not available.  CK initially decreased after rosuvastatin withdrawal, then increased during ezetimibe monotherapy. Pre-treatment CK elevation and absent follow-up after recommended ezetimibe withdrawal prevent attribution to ezetimibe or a specific interaction. |

Abbreviations: CABG, coronary artery bypass grafting; CK, creatine kinase; Lp(a), lipoprotein(a); MI, myocardial infarction; PCI, percutaneous coronary intervention; PCSK9, proprotein convertase subtilisin/kexin type 9; SAMS, statin-associated muscle symptoms.

The assessments are descriptive and are not formal causality scores. Laboratory chronology is presented in Supplementary Table 4. A documented interaction in literature does not establish its contribution in an individual patient. Polypharmacy and shared metabolic or transporter pathways alone were not considered evidence of causal interaction. These observations do not demonstrate a drug–drug–gene interaction.

[1] European Medicines Agency. Brilique (ticagrelor), Summary of Product Characteristics, section 4.5: Rosuvastatin (BCRP substrate). https://www.ema.europa.eu/en/documents/product-information/brilique-epar-product-information_en.pdf (accessed 17 September 2026).
