# Supplementary material for: Liver enzyme elevations during statin–ezetimibe therapy in ABCG2 c.421C>A variant carriers: a case series and narrative review
Source: Front Pharmacol. 2026 Oct 5;17:1840055. doi: 10.3389/fphar.2026.1840055 (PMC13677550; doi:10.3389/fphar.2026.1840055)
Supplement: Supplementary file 3 [file Table3.docx]

Supplementary Table 3. Clinical characteristics, lipid-lowering therapy, laboratory findings, and available pharmacogenetic results in the five comparator patients (C1-C5).

A. Clinical characteristics, therapy, and laboratory findings

| **Patient** | **Sex** | **Age (years)** | **Lipid-lowering therapy** | **Relevant comorbidities / clinical factors** | **Concomitant medications** | **ALT (U/L)** | **AST (U/L)** | **CK (U/L)** |
| --- | --- | --- | --- | --- | --- | --- | --- | --- |
| C1 | M | 47 | Rosuvastatin + ezetimibe (20/10 mg/day) | Coronary artery disease; arterial hypertension; dyslipidemia | Pantoprazole, acetylsalicylic acid, trimetazidine, bisoprolol, diltiazem, perindopril | 23 | 23 | 84 |
| C2 | F | 67 | Rosuvastatin + ezetimibe (40/10 mg/day) | Peripheral arterial disease; arterial hypertension; dyslipidemia; osteoporosis; former smoker | Pantoprazole, rivaroxaban, acetylsalicylic acid, perindopril | 17 | 24 | NR |
| C3 | M | 68 | Rosuvastatin + ezetimibe (40/10 mg/day) | Stable angina; non-obstructive coronary artery disease; arterial hypertension; hyperlipidemia; paroxysmal atrial fibrillation; diaphragmatic hernia | Pantoprazole, bisoprolol, rivaroxaban, ramipril, trimetazidine | NR¹ | NR¹ | NR |
| C4 | F | 63 | Rosuvastatin + ezetimibe (40/10 mg/day) | NSTEMI; multivessel coronary artery disease; arterial hypertension; hyperlipidemia; GERD | Bisoprolol, enoxaparin, acetylsalicylic acid, trimetazidine, pantoprazole, tianeptine, oxazepam, desloratadine | NR¹ | NR¹ | NR |
| C5 | M | 59 | Atorvastatin + ezetimibe (80/10 mg/day) | Ischemic cardiomyopathy (LVEF 40–45%); previous STEMI and in-stent thrombosis; arterial hypertension; hyperlipidemia; left ventricular aneurysm with thrombus; previous TIA and carotid endarterectomy; current smoker | Bisoprolol, acetylsalicylic acid, ticagrelor, perindopril, eplerenone, furosemide, pantoprazole, trimetazidine, warfarin | 42 | 31 | 102 |

Age is reported at blood sampling for pharmacogenetic testing. Laboratory values refer to documented results during combination therapy, not peak values. C1: April 2023; C2: exact date not established; C5: November 2021. ¹ Normal liver enzyme levels during combination therapy were confirmed by review of the medical records, but numerical values were not available. Exact continuous treatment duration was not established for all patients. Concomitant medications reflect the available records; ticagrelor was discontinued in C5 by March 2022.

Abbreviations: ALT, alanine aminotransferase; AST, aspartate aminotransferase; CK, creatine kinase; F, female; GERD, gastroesophageal reflux disease; LVEF, left ventricular ejection fraction; M, male; NR, not reported; NSTEMI, non-ST-elevation myocardial infarction; STEMI, ST-elevation myocardial infarction; TIA, transient ischemic attack.

Supplementary Table 3 continued

B. Available pharmacogenetic results

| **Patient** | ***CYP2C9***  ****2, *3*** | ***CYP2C19***  ****2, *17*** | ***CYP3A4*** | ***CYP3A5***  ****3*** | ***ABCB1***  c.3435C>T | ***ABCC2*** | ***ABCG2***  c.421C>A | ***SLCO1B1*** | ***UGT1A1***  ****28*** | ***UGT2B7***  **c.-161C>T** | ***NAT2***  ****5, *6, *7*** |
| --- | --- | --- | --- | --- | --- | --- | --- | --- | --- | --- | --- |
| C1 | **1/*1* | **2/*17* | **1B: NR *22: *1/*1* | NR | NR | NR | CA | c.388: AG c.463: CC c.1929: AA c.521: TC | NR | NR | **5/*6* |
| C2 | **1/*1* | **1/*17* | **1B: *1/*1 *22: *1/*1* | **3/*3* | CC | NR | CA | c.388: AA c.463: CC c.1929: AA c.521: TT | NR | NR | **4/*6* |
| C3 | NR | NR | NR | NR | NR | NR | CA | c.388: AG c.463: CA c.1929: AA c.521: TT | NR | NR | **5/*6* |
| C4 | **1/*2* | **1/*1* | **1B: *1/*1 *22: *1/*1* | **3/*3* | CT | NR | CA | c.388: AA c.463: CC c.1929: AA c.521: TT | NR | NR | **5/*5* |
| C5 | **1/*1* | **1/*1* | **1B: *1/*1 *22: *1/*22* | **3/*3* | CT | c.-24: CT c.1249: GA | CA | c.388: AG c.463: CC c.1929: AC c.521: TT | *1/*1 | CT | **4/*5* |

NR indicates that no result was reported and was not assumed to represent a reference genotype.

Nucleotide substitutions: *ABCB1* c.3435C>T; *ABCC2* c.-24C>T and c.1249G>A; *ABCG2* c.421C>A; *SLCO1B1* c.388A>G, c.463C>A, c.1929A>C and c.521T>C; *UGT2B7* c.-161C>T. No results were reported for *ABCB1* c.1236C>T or c.2677G>T/A or for *ABCG2* c.34G>A in these five patients.

Abbreviations: ABCB1, ATP-binding cassette subfamily B member 1; ABCC2, ATP-binding cassette subfamily C member 2; ABCG2, ATP-binding cassette subfamily G member 2; CYP2C9, cytochrome P450 family 2 subfamily C member 9; CYP2C19, cytochrome P450 family 2 subfamily C member 19; CYP3A4, cytochrome P450 family 3 subfamily A member 4; CYP3A5, cytochrome P450 family 3 subfamily A member 5; NAT2, N-acetyltransferase 2; SLCO1B1, solute carrier organic anion transporter family member 1B1; UGT1A1, UDP glucuronosyltransferase family 1 member A1; UGT2B7, UDP glucuronosyltransferase family 2 member B7.
