# Supplementary material for: Liver enzyme elevations during statin–ezetimibe therapy in ABCG2 c.421C>A variant carriers: a case series and narrative review
Source: Front Pharmacol. 2026 Oct 5;17:1840055. doi: 10.3389/fphar.2026.1840055 (PMC13677550; doi:10.3389/fphar.2026.1840055)
Supplement: Supplementary file 4 [file Table4.docx]

**Supplementary Table 4.** Laboratory findings before and during statin–ezetimibe therapy and following treatment modification in the 11 patients included in the case series

| **Case** | **Before combination** | **Findings during combination or associated hospitalisation** | **After withdrawal, dose reduction or subsequent treatment** |
| --- | --- | --- | --- |
| **1** | Previous liver enzyme elevations during statin treatment. Immediate pre-combination values NR. | Rosuvastatin 5 + ezetimibe 10; ~3 months. ALT 114; AST 57; GGT 94; CK 1,700. | Aminotransferase elevations persisted during ~13 months of ezetimibe monotherapy. After ezetimibe withdrawal and a treatment-free interval, liver enzymes were documented within reference limits on atorvastatin 10; numerical values NR. |
| **2** | Pre-existing fatty liver/chronic liver disease; previous liver enzyme elevations on statins. Immediate pre-combination values NR. | Rosuvastatin 20 + ezetimibe 10; ~18 months. ALT 120; AST 78; GGT 348. | Enzymes decreased after both drugs were withdrawn and apheresis started; numerical values NR. Combination reintroduced ~1 year later (rosuvastatin 40 + ezetimibe 10). Four years later: ALT 140; AST 184; GGT 375. |
| **3** | CK approximately 1,000 U/L during prior rosuvastatin treatment; liver enzyme and CK values immediately before ezetimibe addition NR. | Rosuvastatin 20 + ezetimibe 10; ~12 months. ALT 91; AST 60; CK 1,069. | Switched to atorvastatin 40 mg/day + ezetimibe 10 mg every other day. CK fluctuated but gradually improved; numerical follow-up values NR. |
| **4** | Baseline liver enzymes NR | Atorvastatin 40 + ezetimibe 10. 18 Jul 2022: ALT 123; AST 213; ALP 116; GGT 535. 7 Sep 2022: ALT 93; AST 219; ALP 167; GGT 1,048. | Atorvastatin withdrawn; ezetimibe subsequently replaced with alirocumab. Follow-up: ALT 98; AST 80; GGT 419. Exact sampling date and regimen at sampling could not be established. |
| **5** | On rosuvastatin 40: ALT 31; AST 26; GGT 38. | Rosuvastatin 40 + ezetimibe 10; ~7–8 months. ALT 93; AST 57; ALP 81; GGT 55. | Ezetimibe withdrawn; rosuvastatin reduced to 20. ~1 month later (9 Oct 2023): ALT 54; AST 39; ALP 76; GGT 44; bilirubin 5.1; CK 74. Later on atorvastatin 80: ALT 57; AST 39; GGT 157. Normalisation documented after switch to alirocumab; numerical values NR. |
| **6** | Baseline liver enzymes NR | Rosuvastatin 20 + ezetimibe 10; ~3 months. Serial results: ALT 161 → 204; AST 69 → 79; GGT 24 → 23. | Rosuvastatin withdrawn; ezetimibe continued. Evolocumab added ~1 year later. Follow-up laboratory values NR. |
| **7** | Baseline liver enzymes and CK NR | Liver enzymes and CK documented within reference limits on rosuvastatin 20 + ezetimibe 10 despite myalgia. Later on fluvastatin 80 + ezetimibe 10: ALT 53; AST 25; GGT 37. | Ezetimibe reduced to every other day, then every third day; fluvastatin continued. Follow-up laboratory values NR. |
| **8** | Baseline liver enzymes and CK NR. | Rosuvastatin increased to 40 + ezetimibe 10; hospitalisation in 2024. Admission: ALT 323; AST 517; GGT 50. Hospital peaks: ALT 773; AST 1,206; CK 11,837. | Both drugs withdrawn during hospitalisation. The interval between withdrawal and peak results could not be established; subsequent laboratory recovery was not documented in the reviewed records. |
| **9** | Baseline liver enzymes and CK NR | Rosuvastatin + ezetimibe; doses NR. Highest recorded ALT 239; CK 6,704. Additional AST 71. Another result: ALT 239; GGT 23; CK 1,760. Sampling dates and timing relative to withdrawal could not be established. | Ezetimibe 10 monotherapy after combination withdrawal. Follow-up laboratory values NR. |
| **10** | Baseline liver enzymes NR | Rosuvastatin 20 + ezetimibe 10; 2 months. ALT 133; AST 49; GGT 55. | Rosuvastatin reduced to 10; ezetimibe continued. After 3 months: ALT 76; AST 42; GGT 46. Further reduction to rosuvastatin 5 implemented; subsequent laboratory values NR. |
| **11** | CK 1,700 in Mar 2024, ~10 months before combination; immediate pretreatment values NR. | Rosuvastatin 10 + ezetimibe 10. Oct 2025: ALT 69; AST 62; CK 1,244. | Rosuvastatin withdrawn; ezetimibe 10 continued. Nov 2025: CK 738. Jan 2026: ALT 83; AST 148; CK 5,466. Ezetimibe withdrawal recommended; withdrawal and subsequent laboratory results were not documented in the reviewed records. |

Qualitative improvement or normalisation is reported where documented; no missing numerical values were imputed. Results and peak values may represent different sampling dates. Subsequent treatment changes do not necessarily represent formal rechallenges.

Abbreviations: ALP, alkaline phosphatase; ALT, alanine aminotransferase; AST, aspartate aminotransferase; CK, creatine kinase; GGT, gamma-glutamyl transferase; NR, not reported in the clinical documentation available for this case series.

Units: Enzyme activities are expressed in U/L and total bilirubin in µmol/L. Drug doses are expressed in mg/day unless otherwise stated.
